# The impact of the COVID-19 pandemic on the mental health of medical staff considering the interplay of pandemic burden and psychosocial resources—A rapid systematic review

**DOI:** 10.1371/journal.pone.0264290

**Published:** 2022-02-22

**Authors:** Julian Hannemann, Alan Abdalrahman, Yesim Erim, Eva Morawa, Lucia Jerg-Bretzke, Petra Beschoner, Franziska Geiser, Nina Hiebel, Kerstin Weidner, Susann Steudte-Schmiedgen, Christian Albus

**Affiliations:** 1 Department of Psychosomatics and Psychotherapy, University Hospital Cologne, Cologne, Germany; 2 Department of Psychosomatic Medicine and Psychotherapy, University Hospital of Erlangen, Friedrich-Alexander University Erlangen-Nürnberg (FAU), Erlangen, Germany; 3 Department of Psychosomatic Medicine and Psychotherapy, Ulm University Medical Center, Ulm, Germany; 4 Department of Psychosomatic Medicine and Psychotherapy, University Hospital of Bonn, Bonn, Germany; 5 Department for Psychotherapy and Psychosomatic Medicine, Faculty of Medicine, Technische Universität Dresden, Dresden, Germany; Medical University of Vienna, AUSTRIA

## Abstract

**Background:**

In times of the global corona pandemic health care workers (HCWs) fight the disease at the frontline of healthcare services and are confronted with an exacerbated load of pandemic burden. Psychosocial resources are thought to buffer adverse effects of pandemic stressors on mental health. This rapid review summarizes evidence on the specific interplay of pandemic burden and psychosocial resources with regard to the mental health of HCWs during the COVID-19 pandemic. The goal was to derive potential starting points for supportive interventions.

**Methods:**

We conducted a rapid systematic review following the recommendations of the Cochrane Rapid Reviews Methods Group. We searched 7 databases in February 2021 and included peer-reviewed quantitative studies, that reported related data on pandemic stressors, psychosocial resources, and mental health of HCWs.

**Results:**

46 reports were finally included in the review and reported data on all three outcomes at hand. Most studies (n = 41) applied a cross-sectional design. Our results suggest that there are several statistically significant pandemic risk factors for mental health problems in HCWs such as high risk and fear of infection, while resilience, active and emotion-focused coping strategies as well as social support can be considered beneficial when protecting different aspects of mental health in HCWs during the COVID-19 pandemic. Evidence for patterns of interaction between outcomes were found in the context of coping style when facing specific pandemic stressors.

**Conclusions:**

Our results indicate that several psychosocial resources may play an important role in buffering adverse effects of pandemic burden on the mental health of HCWs in the context of the COVID-19 pandemic. Nevertheless, causal interpretations of mentioned associations are inadequate due to the overall low study quality and the dominance of cross-sectional study designs. Prospective longitudinal studies are required to elucidate the missing links.

## Introduction

Since the coronavirus outbreak in Wuhan in December 2019, the epidemic has quickly become a global threat. On January 30^th^, 2020, the World Health Organization (WHO) declared a "health emergency of international concern" and classified the spreading of the COVID-19 disease as a pandemic on March 11^th^, 2020. As of July 1^st^, 2021, more than 184 million cases and 3.95 million deaths have been registered worldwide. By October 2021 there are more than 94.000 deaths by or in association with the coronavirus in Germany [1]. The SARS-CoV-2 virus is a beta coronavirus and mainly transmitted by droplet infection, aerosols, and direct contact with infected people. The clinical symptoms of COVID-19 disease are comparable to influenza and include dry cough, fever, disorders of the sense of smell or taste, and pneumonia [2]. Older patients and patients with pre-existing medical conditions are at particular risk of severe disease progression. Repeated mutations have resulted in subtypes which, according to the current status, differ mainly in their infectivity [3].

In times of a global pandemic healthcare workers (HCWs) fight the disease at the frontline ensuring the care of covid-19 infected and otherwise sick patients which leaves them–despite protective clothing–at a 3 to 4-fold increased risk of being infected with the virus themselves [4]. Due to high levels of incidence, numbers of intensive care treatments with respect COVID-19 infections are increasing accordingly. As a consequence, hospital resources have to be reorganized (e.g., postponing elective treatments) while workload increases significantly [5, 6]. Overall, the COVID-19 pandemic places an additional physical and mental burden on all front and second-line HCWs irrespective of their level of exposure to potentially Sars-CoV-2 infected patients. Published data from previous pandemic outbreaks such as the SARS outbreak in 2003 [7, 8], the H1N1 pandemic in 2009 [9, 10], and the Ebola outbreak 2014 [11] have shown that HCWs are at increased risk for symptoms of mental disease such as post-traumatic stress disorder, depression, states of anxiety and fatigue when exposed to pandemic stressors, such as anxiety of falling ill or infecting others, being avoided by others (stigmatization), feeling stressed at work [8], lack of knowledge about infectivity/virulence and emotional exhaustion [10].

Several potential pandemic stressors for HCWs in the current pandemic have been identified. These include having a higher COVID-19 contraction risk (poorer health, contact with COVID-19 patients, working in high-risk areas), social isolation, spending longer time watching COVID-19 related news [10], being concerned about personal health and infecting family members [12]. The authors of this paper are involved in a research group on the mental health of HCWs in German University Hospitals, the VOICE study. Multiple regression analysis on data generated by the VOICE study group revealed that insufficient recovery during leisure time, increased alcohol consumption, and less trust in colleagues in difficult situations at work are statistically associated with elevated symptoms of depression. Meanwhile the increased fear of becoming infected with COVID-19 was positively related to symptoms of anxiety [13]. Overall HCWs showed significantly elevated levels of anxiety and depression when compared to pre-pandemic data from the general German population. Furthermore, there was a statistically significant relation between clinically relevant levels of depressiveness and the reduced willingness to vaccinate against Sars-CoV-2 [14]. Our data also revealed that levels of generalized anxiety and depressiveness, increased fear of infecting relatives, as well as medical profession (MTA compared to physicians) were the most relevant statistical predictors for symptoms of post-traumatic stress disorder (PTSD) in HCWs [15]. In times of increased physical and mental burden HCWs have to rely on psychosocial resources potentially mitigating the contradicting effects of pandemic stressors on mental health. Resilience and coping strategies have already shown to be protective factors regarding the development of symptoms of PTSD. This includes the use of humor, altruistic acceptance of the risks associated with work and the maintenance of trusting relationships [16].

Recently published data from our study group on 7765 HCWs in Germany revealed that elevated levels of perceived social support and optimism were negatively associated with symptoms of anxiety and depression [17]. This association seemed to be stronger than the effect of sociodemographic and occupational factors such as female gender or contact with COVID-19 infected patients. Furthermore, we were able to show that higher sense of coherence was strongly related to less symptoms of anxiety and depression in HCWs [18]. Sufficient social support seems to reduce the occurrence of anxiety symptoms through positive coping strategies and the reduction of negative coping behavior [19]. Another study focused on resilience and defense mechanisms as psychosocial resources and found that both resilience and adaptive defense mechanism may protect individuals from severe stress and burnout symptoms [20].

Even though several risk and protective factors for mental health of HCWs during pandemic outbreaks have been identified, evidence regarding specific interactions and mediating mechanisms is scarce. This information is nevertheless crucial for developing preventive and efficient interventions during the current and potential future pandemics. This paper aims to systematically review available studies on the interplay between psychosocial resources and pandemic burden with regard to mental health outcomes in HCWs. Based on our findings we will report on implications for psychosocial interventions and future research.

## Methods

This rapid systematic review followed the recommendations of the Cochrane Rapid Reviews Methods Group [21]. Stakeholders were the directors or collaborators in leading positions of psychosomatic departments in five university hospitals in Germany. Research question and search strategy were discussed and consented within this study group. After extensive literature search and first screening of records within title and abstracts by one researcher, two reviewers independently screened all included full-text articles and resolved conflicts by discussion using the Rayyan online tool for systematic reviews [22]. The review was registered at PROSPERO (CRD42021242035). Searches were conducted on 4th of February 2021 on the following databases:

- PubMed,
- Web of Science (Core Collection),
- MEDLINE (via EBESCOhost),
- PsycArticles (via EBESCOhost),
- PSYNDEX (via EBESCOhost),
- PsycINFO (via EBESCOhost) and
- Cochrane Library.

Initially, we defined 5 search term clusters following the P(I)CO criteria (“I” for intervention was left out since it is not applicable to our research question). The first cluster addressed the population (P) aspect of the research question which was “healthcare workers” and comprised 46 synonyms combined by “or” as a Boolean. As of the condition (C) aspect we identified 11 terms to describe “Covid-19” also combined by the “or” Boolean within the parentheses. The third cluster referred to the main outcome (O) “mental health” including 26 synonyms and relating constructs such as “burnout” or “depression”. The fourth and the fifth cluster comprised terms related to “pandemic burden” (23 terms) and “psychosocial resources” (11 terms). During the trial search it became apparent, that some studies did not handle their constructs related to pandemic burden and mental health selectively in terms of indexing. To ensure a more complete view of the existing literature, we decided to integrate those two clusters within one. The remaining four clusters were combined by the Boolean “and”. There were no restrictions on search term fields (e.g., abstract, title, etc.) except for the Web of Science database, where the total number of search terms in an “All Fields query” must not exceed 100. Therefore, the “Topic (title, abstract, author keywords, and Keywords Plus) query” was used with Web of Science. Ultimately, restrictions were set in terms of “peer-review only” (EbescoHost only), language (German/English) and publication year (2019–2021). Furthermore, references of identified systematic reviews and included publications were screened for studies relevant to the research question. For a full report of final search terms see S1 Appendix.

### Types of participants

The target population was healthcare personnel in hospitals or communities such as physicians, nursing staff, paramedics, ambulance personnel, psychologists, clinical medical students, therapists (e.g., physiotherapists) and other hospital staff (e.g., medical-technical staff in laboratories or pharmacy, ward clerk, administrative staff, etc.) that worked in their medical facility during the COVID-19 pandemic irrespective of their level of exposure to COVID-19 infected patients. Studies which exclusively sample any other population than the one mentioned above (e.g., general population, patients, or non-clinical medical students) or did not provide a subgroup analysis on HCWs were excluded.

### Types of studies included

Empirical quantitative studies with the following study designs were included: cohort, case-control, prospective, and cross-sectional studies. Included studies must contain at least one standardized and validated measurement of mental health, one measurement of pandemic burden and one measurement of psychosocial resources. Additionally, factors of all three outcome domains had to be integrated into a mutual statistical model and therefore put in statistical relation to each other (e.g., via structural equation modelling (SEM) or hierarchical linear regression analysis). This focus on the specific interplay of factors of pandemic burden and psychosocial resources concerning mental health in medical professionals is the most prominent distinctive factor from similar reviews.

The following types of publications were excluded:

- Qualitative survey studies
- Intervention studies
- Editorials, letters and conference papers
- “grey literature” such as conference abstracts and dissertations
- No full-text available
- Not peer-reviewed
- Reviews and meta-analysis (which were screened for relevant references before exclusion)

### Data extraction and quality assessment

Data of included studies was extracted by two reviewers (first and second author of this paper) via Microsoft Excel [23]. Data extraction included information on authors (abbreviated by “et al.” if there are more than two authors), year of publication, country, study design, participants (including sociodemographic data such as gender and age) and sample size, assessed outcomes and instruments used, main results relevant to the research question and annotations. Study quality was assessed by the Newcastle—Ottawa Quality Assessment Scale (NOS) [24], which was also adapted for cross-sectional studies where appropriate [25]. The NOS works with a star-rating system, which is an efficient and economical way to conduct quality assessment, especially when one is working within limited resources. The total amount of given stars for all three criteria (“selection”, “comparability” and “outcome”) determines overall study quality. For cohort studies there was a maximum of 9 stars while the adapted version for cross-sectional studies indicated a maximum of 10 stars. The cut-off between low and moderate study quality was 5 stars within both scales. With respect to high study quality, we chose a score of 7 stars or higher for cohort studies and a score of 8 or higher with cross-sectional reports. Studies were rated by two independent reviewers and conflicts resolved by discussion.

### Data synthesis and analysis

Since data of included reports was heterogeneous in terms of outcome measures, population, country, pandemic status, etc. we decided to use a tabular and written narrative approach to data synthesis. Goals were the presentation of complex data and the preliminary identification of general patterns within variables.

## Results

**Fig 1:**
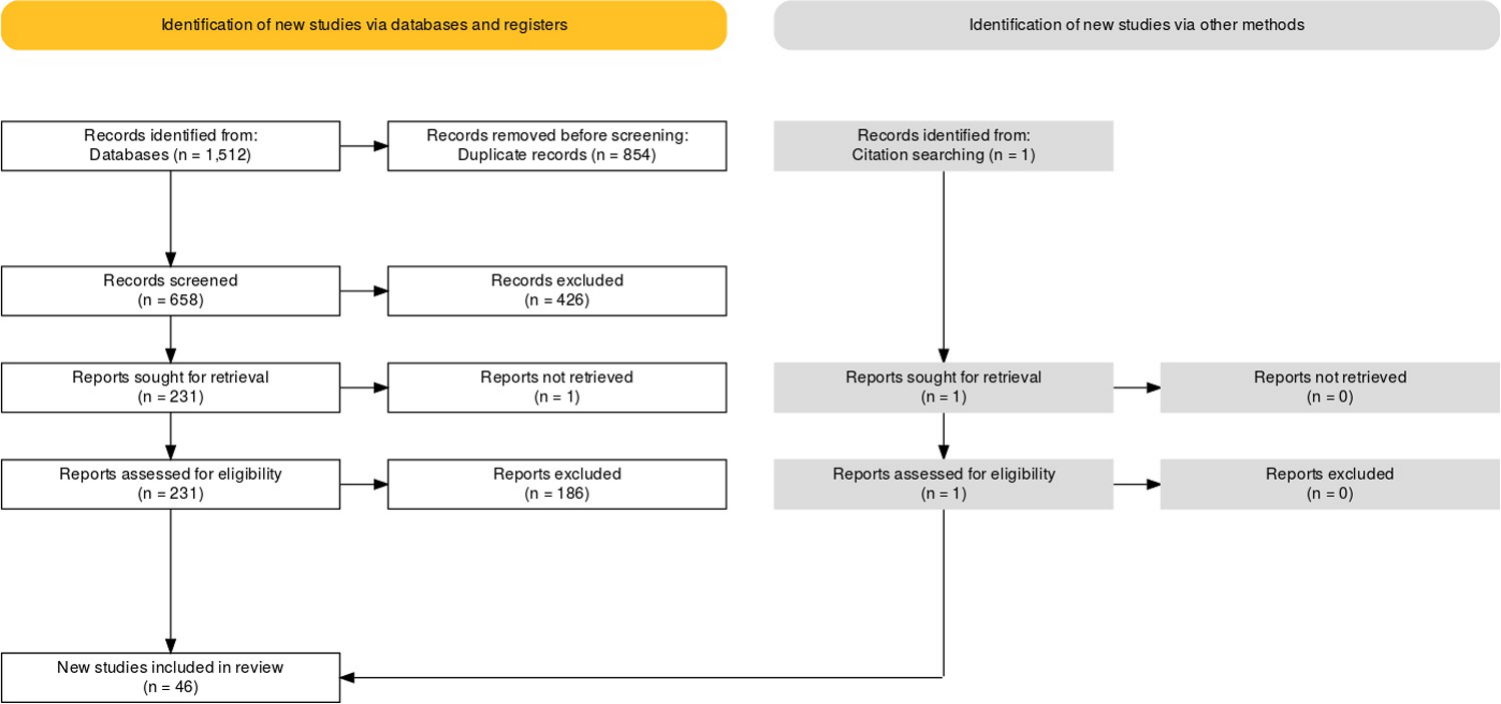
PRISMA flow diagram.

As presented in Fig 1 we identified 1512 records in all databases. Within the review process 1467 reports were excluded while one additional study was identified by citation searching within full-text screening, resulting in a total number of 46 reports that were finally included in the review.

### Study characteristics

As seen in Table 1 the vast majority of included reports applied a cross-sectional study design (n = 41), while there were only four studies that generated longitudinal data. One study used a mixed-method approach, but only the quantitative cross-sectional analysis was integrated in this review. Not surprisingly most studies (n = 13) were conducted in China, where the coronavirus first emerged in the end of 2019, followed by Italy (n = 5), USA (n = 4), Spain (n = 4), Turkey (n = 4) and thirteen other countries (n = 16). Sample sizes ranged between 96 and 7124 participants. In terms of gender there was a clear surplus of female participants ranging from 39.2–100%. Only two studies surveyed less than 50% female participants while twelve records reported female gender rates of 80% and above. Most studies showed low to moderate study quality.

**Table 1:** Characteristics of included studies.

Authors	Year of p	Country	Study design	Population (sample size)	Gender/Age/Years of experience	Relevant measures of mental health	Relevant measures of pandemic burden	Relevant measures of psychosocial resources	Study quality
**Balay-Odao et al.**	2021	Saudi Arabia	cross-sectional	**Nurses (n = 281)**	**• Gender**: **•** Female = 241 (85.8%) **•** Male = 40 (14.2%) **• Age Ø (range)**: 33.25 ± 6.38 **• Years of experience Ø (range)**: 10.66 ± 6.37	Depression & Anxiety (via DASS-21)	Hospital Preparedness Assessment Tool	Resilience (via Resilience Scale for Nurses)	High
**Bettinsoli et al.**	2020	Italy	cross-sectional	**HCW (n = 580)** **•** Physicians (n = 413, 71%) **•** Nurses (n = 121, 21%) **•** Staff (n = 46, 8%)	**• Gender:** **•** Female = 341 (40%) Male = 232 (59%) **• Age Ø (range):** NA **• Years of experience Ø (range):** NA	Mental well-being (via GHQ-12)	Separation distress (via ASA); Emotional Symptoms (via SDQ); Emotional Dysregulation (via DERS-18); Contextual variables (e.g., living in high-risk area via self-constructed items)	Resilience (via BRCS), Coping Self-Efficacy (via CSES)	Moderate
**Britt et al.**	2021	USA	longitudinal	**Emergency medicine personnel (n = 97)** **•** Physicians (n = 56) **•** Advanced practice providers (n = 26) **•** residents (n = 15)	**• Gender**: Female = 39.2% Male = 60.8% **• Age Ø (range)**: 40 (27–70) **• Years of experience Ø (range):** NA	Mental Health Strain (via PWBI)	COVID-19 Work and Personal Demands (via self-constructed items based on prior research); Hours worked (via emergency shift administration database)	social support and meaningful work (via self-constructed items based on prior research)	Low
**Bruffaerts et al.**	2021	Belgium	cross-sectional	**HCW (n = 6409)**	• **Gender**: Female = 72.4% Male = 27.6% **• Age Ø (range):** 41.6 **• Years of experience Ø (range):** NA	Suicidal thoughts and behaviors (via C-SSRS)	Generalized anxiety disorder (via GAD-7); Major depressive disorder (via PHQ-9); post-traumatic stress disorder (via PCL-5); Panic attacks (via number of self-reported panic attacks), exposure to COVID19; work-related factors	Social support (via two items "living together" and "social network available)	Moderate
**Chen et al.**	2021	China	cross-sectional	**HCW (n = 902)** **•** Physicians (n = 543) **•** Nurses (n = 311) **•** Others: administrative and management staff (n = 48)	**• Gender**: Female = 68.63% Male = 31.37% **• Age Ø (range):** NA **• Years of experience Ø (range)**: NA	Generalized anxiety disorder (via GAD-7); Depression symptoms (via PHQ-9)	Workload, health condition, specific tests related to COVID-19 (via structured questionnaire), Job burnout (via CMBI)	Coping style (via TCSQ)	Moderate
**Chew et al.**	2020	Singapore	longitudinal	**T1: Residents (n = 274)** • medical disciplines (n = 191) • Surgical disciplines (n = 83) **T2: Residents (n = 221)** • medical disciplines (n = 167) • Surgical disciplines (n = 53)	**• Gender**: Female = T1: 51.5%; T2: 49.8% Male = T1: 48.5%; T2: 50.2% **• Age Ø (range)**: T1: 30.6; T2: 30.8 **• Years of experience** Ø (range): T1: 6.07; T2: 6.12	Traumatic stress (via IES-R)	Perceived Stress Scale (via PSS), Healthcare Workers Stigma Scale (via HWSS), deployment outside of one’s usual job scope, deployment to high-risk areas (NCID), Exposed to patients with respiratory illness	Coping (via COPE)	Low
**Chow et al.**	2021	Malaysia	cross-sectional	**HCW (n = 200)** • medical doctor (n = 139) nurses (n = 50) • assistant medical officer (n = 7) • clinical attendants (n = 2) • non-clinicians (n = 2)	• **Gender:** Female = 60.5% Male = 39.5% • **Age Ø (range):** 20–30: 25% 31–40: 70.5% 41–50: 3.5% >51: 0.5% • **Years of experience Ø (range):** NA	Anxiety and Depression (via HADS-M)	negative religious coping (via BRCOPE-M), all participants managed patients suspected to have or infected with COVID-19	positive religious coping (via BRCOPE-M)	Moderate
**Correia & Almeida**	2020	Portugal	cross-sectional	**HCW (n = 497)** • physicians (n = 229) • nurses (n = 268)	• **Gender:** Female = physicians: 52%; nurses: 73% Male = physicians: 48%; nurses: 27% • **Age Ø (range):** physicians: 36.54 (23–70) nurses: 34.96 (22–69) • **Years of experience Ø (range):** NA	Burnout (via OLBI)	Workload (via 1-Item from AWS), COVID-specific factors (via 3 self-constructed items)	Empathy (via BES-A), Meaningful Work (via 2 Items from WAMI), Justice perceptions (via 2 self-constructed items)	Moderate
**Dong et al.**	2020	China	cross-sectional	**HCW (n = 4618)** • nurses (n = 2889) • physicians (n = 1138) • technicians (n = 319) • health • administrators (n = 272)	• **Gender**: Female = 86.7% Male = 16.3% • **Age Ø (range):** ≤ 29 = 34.6% 30–39 = 41.3% 40–49 = 16.8% ≥50 = 6.9% • **Years of experience Ø (range):** 12.19 (<1 - >50)	Emotional distress (anxiety, depression, and/or suicidal ideation via HEI)	Exposure to COVID-19 (via self-constructed items), perceived risk of COVID-19 (via self-constructed items)	Family relationships (via self-constructed items)	Moderate
**Hines et al.**	2020	USA	longitudinal survey	**HCW (n = 96)** • attending physician (n = 60, 62.5%), • fellow physician (n = 14, 14.6%), • resident physician (n = 12, 12.5%), • other (n = 10, 10.3%)	• **Gender**: • Female = 49 (51.0%) • Male = 47 (49.0%) • **Age Ø (range):** 40.6 ± 10.4 • **Years of experience Ø (range)**: 14.0 ± 10.3	Moral injury (via MIES), psychological distress (via IES-R)	psychological distress (via IES-R), stressful work environment	Resilience (via self-constructed questionnaire)	Moderate
**Huang et al.**	2020	China	cross-sectional	**HCWs (n = 364)** • nurses (n = 119, 32.7%) • technicians (n = 245, 67.3%)	• **Gender**: • Female = 214 (58.8%) • Male = 150 (41.2%) • **Age Ø (range):** 32 years (27–40 years) • **Years of experience Ø (range):** 10 years (5–19)	Anxiety (via SAS)	Presence of suspected symptoms in participants, contact with confirmed cases, fear of an uncontrollable epidemic and inability to pay rent or mortgage (via self-constructed items)	Resilience (via CD-RISC)	Moderate
**Jokic-Begic, Korajlija, Begic**	2020	Croatia	cross-sectional	**Physicians (n = 725)** • psychiatrists (22.8%), • internal medicine specialties (37.4%) • surgical specialization (14.2%) • family physician (8.0%), • anesthesiologists (5.7%) • infectologists and epidemiologists (2.3%) • emergency physicians (1.9%) • physicians of other specializations (7.4%)	• **Gender**: Female = 522 (72%) Male = 203 (18%) • **Age Ø (range):** 48.3 (± 11.26), 26–81 years • **Years of experience Ø (range):** NA	Psychological distress such as well-being, symptoms/problems, functioning and risk (via CORE-YP)	COVID-19 anxiety (via CAS) and pandemic concerns (via COVID-19 pandemic concerns measure)	Coping strategies (self-constructed items), life satisfaction (self-constructed single item), resilience (via BRS), psychological flexibility (via AAQ-II)	Moderate
**Kim et al.**	2020	USA	cross-sectional	**Nurses (n = 320)**	• **Gender**: Female = 302 (94.4%) Male = 18 (5.6%) • **Age Ø (range):** 33 years (21–67) • **Years of experience Ø (range):** 10.1 (0–44)	Stress (via PSS), Anxiety (via GAD-7), Depression (via PHQ-9)	COVID-19 patient care, work hours, quarantine (via self-constructed items)	Resilience (via CD-RISC), social support such as Adaptation, Partnership, Growth, Affection and Resolve (via Family APGAR), spiritually support (via 12-Item Spiritually Support Scale)	Moderate
**Krammer et al.**	2020 (runs on)	Switzerland	longitudinal study (cross-sectional analysis)	**HCWs (n = 100)** • physicians (n = 18) • nurses (n = 41) • psychology / pedagogy (n = 10) • administration (n = 23) • emergency (n = 4) • midwife (n = 1) • physiotherapist (n = 3)	• **Gender**: Female = 74 (74.0%) Male = 26 (26.0%) **• Age Ø (range)**: 42.6 ± 13,5 (16–66) • **Years of experience Ø NA (range):** NA	Adjustment disorder symptoms (via ADNM-20), Depression symptoms (via PHQ-9)	Perceived stress symptoms and worries through COVID-19 (via self-constructed items), prior traumatic experiences (via CIDI List, ACE)	Coping strategies (via SCI)	Moderate
**Krok, Zarzycka**	2020	Poland	cross-sectional	**HCWs (n = 226)** • doctors (n = 51) • nurses (n = 113) • laboratory technicians (n = 22) • aides and assistants (n = 29) • physiotherapists (n = 11)	• **Gender**: Female = 58.8% Male = 41.2% • **Age Ø (range):** 37.36 ±13.59 • **Years of experience Ø (range):** NA	Psychological well-being (via psychological well-being Scale)	Fear, perceived threat, and risk of contracting COVID-19 (via self-constructed items)	problem focused- and emotional Coping, Meaning in Life (via 10-item Meaning in life questionnaire), Existential Mattering (via self-constructed items)	Low
**Liao et al.**	2020	China	cross-sectional	**clinical nurses (n = 1092)**	• **Gender**: Female = 1043 (99.51%) Male = 49 (4.49%) • **Age Ø (range)**: NA, (20 - >49) • **Years of experience Ø NA (range)**: NA	Acute stress disorder (ASD) symptoms (via SASRQ)	Working in epidemic department of the hospital	self-efficacy (via GSES) and perceived social support (via PSSS)	Moderate
**Li et al.**	2020	China	cross-sectional	**Public health workers (n = 6317)** • CDC workers (from Centers for Disease Control and Prevention, n = 2,313) • PHI (from primary health care institutes, n = 4,004)	• **Gender**: Female = 64.6% Male = 35.4% • **Age Ø (range)**: 38.7 ± 9.43 • **Years of experience Ø NA (range):** NA	Poor perceived health, Depression (via PHQ-9), Anxiety (via GAD)	Perceived troubles at work, Perceptions related to COVID-19 and work (via self-constructed items)	Perceived support (via self-constructed items)	Moderate
**Liu et al.**	2020	China	cross-sectional	**Nurses (n = 1364)** • frontline nurses (n = 568) • second-line nurses (n = 706)	• **Gender**: Female = 1,072 (79%) Male = 292 (21%) • **Age Ø (range)**: 30.0 (27–34) • **Years of experience Ø (range)**: 8 years (4–12)	Psychological distress (via GHQ-28)	Anxiety about COVID-19 pandemic, sources of information and degree of concern about the epidemic (via self-constructed items)	Social support, coping strategies (via self-constructed items)	Moderate
**Li, Zhou, Xu**	2020	China	predictive cohort study	**Nurses (n = 356)** • Primary RNs (registered nurses) (n = 59, 16.6%) • Nurse practitioners (n = 48, 13.5%) • senior nurses (n = 133, 37.4%) • Nurse managers / supervisors (n = 74, 20.8%) • deputy chief nurse (n = 42, 11.8%)	• **Gender**: Female = 307 (86.2%) Male = 49 (13.8%) • **Age Ø (range):** 31.3 (NA) • **Years of experience Ø (range)**: <2 - >20	PTSD symptoms (via PCL-5)	Stress level (via PSS)	Resilience (via CD-RISC)	Low
**Lorente, Vera, Peiró**	2020	Spain	cross-sectional	**Nurses (n = 421)**	• **Gender**: Female = 93.6% Male = 6.4% • **Age Ø (range):** 36 years ± 10.4 • **Years of experience Ø (range)**: 12 years ± 10.1	Psychological distress (anxiety, depression, stress, via DASS-21)	Stressors (Work overload, Insufficient preparation, Lack of support, Death and Dying, Fear of Infection, via NSS)	Coping strategies: PFC (problem-focused), EFC (emotion-focused) (via Brief COPE), Resilience (via Resilience scale by Stephens et al. (2013))	Low
**Luceño-Moreno et al.**	2020	Spain	cross-sectional	**HCWs (n = 1422)**	• **Gender**: Female = 1228 (86.4%) Male = 194 (13.6%) • **Age Ø (range):** 43.88 ± 10.82 (19–68) • **Years of experience Ø (range)**: NA	Anxiety and Depression (via HADS), Posttraumatic stress (via IES-R)	Burnout subscales (via MBI-HSS), variables specific to COVID-19	Resilience (via BRS)	Moderate
**Manzano García et al.**	2020	Spain	cross-sectional	**Nurses (n = 771)**	• **Gender**: Female = 6940 (90%) Male = 77 (10%) • **Age Ø (range):** 42.38 ± 11.42 (21–65) • **Years of experience Ø (range)**: 17.09 ± 6.67 (1–43)	Burnout (via CESQT)	Role conflict, role ambiguity and work overload (via UNIPSICO Battery), perceived threat of COVID-19 (via Scale of perceived threat of COVID-19)	Social support & autonomy (via UNIPSICO Battery)	High
**Mosheva et al.**	2020	Israel	cross-sectional	**Physicians (n = 1106)**	• **Gender**: Female = 542 (49%) Male = 564 (51%) • **Age Ø (range):** 46.07 ± 13.20 (25–88) • **Years of experience Ø (range):** NA	Anxiety (via PROMIS)	Pandemic‐related stress factors (via PRSF)	Resilience (via CD-RISC)	Moderate
**Mo et al.**	2020	China	cross-sectional	**Nurses (n = 200)**	• **Gender**: Female = 178 (89.0%) Male = 22 (11%) • **Age Ø (range)**: 32.12 ± 7.65 (21–48) • **Years of experience Ø (range)**: 7.89 ± 5.68 (2–32)	Anxiety (via SAS)	Work stress and overload (via SOS)	Self-efficacy (via GSES)	Moderate
**Nie et al.**	2020	China	cross-sectional	**Nurses (n = 263)**	• **Gender**: Female = 202 (76.7%) Male = 61 (23.3%) • **Age Ø (range)**: 89.7% were younger than 39 years (<30–59) • **Years of experience Ø (range):** NA (<1 - >10)	Psychological distress (anxiety or depression, loss of self-confidence and inability to make decision via GHQ-12)	COVID-19 related stress symptoms such as intrusion, arousal, avoidance (via COVID-19 adapted IES-R), working overtime, concern for own and family	Social support (via PSSS), Coping strategies (via SCSQ)	Moderate
**Ni et al.**	2020	China	cross-sectional	**Participants (n = 1791)** • community based (n = 1577) • **HCW (n = 214)**	• **Gender: HCW** Female = 68.8% Male = 31.2% • **Age Ø (range):** 18–34: 58.9% 35–44: 33.6% 45 or above: 7.5% • **Years of experience Ø (range):** NA	Depression (via PHQ-2), Anxiety (via GAD-2)	Daily time spent on COVID-19 news on TV or Social Media, Confirmed close contact with COVID-19,	Social Support (via MOS-SSS)	Moderate
**Orrù et al.**	2020	Italy (participants from 45 countries are involved)	cross-sectional	**HCW (n = 184)** in 45 countries • physicians (n = 138, 75.0%) • surgeons (n = 3, 1.6%) • nurses (10, 5.4%) • psychologists (n = 2, 1.1%) • other health professionals (n = 31, 16.8%)"	• **Gender**: Female = 50.5% Male = 48.9% • **Age Ø (range):** 46.45 ± 11.02 (24–74) • **Years of experience Ø (range):** Seniority 19.90 ± 11.58 (0–50)	Secondary traumatic stress (via STSS)	Burnout (via MBI-HSS), perceived Stress (via PSS with Subscales for Intrusion, Avoidance and Arousal), personal and professional experiences during COVID-19 (via self-constructed items)	Self-efficacy (via GSE), Resilience (RS-14)	Moderate
**Özdemir, Kerse**	2020	Turkey	cross-sectional	**HCW (n = 169)** **•** emergency medicine/laboratory/x-ray technicians (39.1%) **•** paramedics (26%) **•** nurses (15.4%) **•** health officers (10.1%), • health care workers from other professions (9.5%)	• **Gender**: Female = 58.6% Male = 41.4% • **Age Ø (range):** NA • **Years of experience Ø (range):** 78.7% had 5 or more years of experience	Emotional Exhaustion as measure of burnout (via MBI)	Job stress (via JSS)	Optimism (via OPS)	Low
**Pang et al.**	2020	China	cross-sectional	**Nurses (n = 282)**	• **Gender**: Female = 250 (88.65%) Male = 32 (11.348%) • **Age Ø (range):** 31.61 ± 7.60 (20–55) • **Years of experience Ø NA (range):** NA	Depression (via PHQ-9), Anxiety (via GAD-7)	lack of sleep, working overtime	Resilience (via CD-RISC), coping styles (via SCSQ)	Moderate
**Ramaci et al.**	2020	Italy	cross-sectional	**HCW (n = 273)** • doctors (n = 206) • nurses (n = 67)	• **Gender**: Female = 137 Male = 136 • **Age Ø (range):** 46.67 ± 8.36 (NA) • **Years of experience Ø (range):** 13.32 years ± 10.7 (NA)	Compassion satisfaction, Burnout, Compassion fatigue (via ProQOL)	high work pressure demands relating to mental load, unfavorable demands of the physical environment (via JCQ); Stigma Discrimination / Fear (via self-administered MC-questionnaire)	Self-efficacy/self-esteem (via RSES)	Moderate
**Roslan et al.**	2020	Malaysia	mix method, cross sectional and qualitative interviews	**HCW (n = 933)** • physicians (n = 203, 22.7%) • social workers (n = 128, 14.3%) • assistant medical officer (n = 120, 13.4%) • Nurse (n = 47, 5.3%) • Other: Psychologist, Pharmacist, Food preparation personal (n = 435, 46%)	• **Gender**: Female = NA Male = NA • **Age Ø (range)**: Less than 40 years (n = 682, 76.4%) 40 years and above (n = 211, 23.6%) • **Years of experience Ø (range)**: NA	Burnout (via CBI)	Perceived Inadequate psychosocial support received at work, suffering from some medical illness, to work overtime	psychosocial support at work, spiritual routines	Moderate
**Seçer, Ulas, Karaman-Özlü**	2020	Turkey	cross-sectional	**HCW (n = 390)**	• **Gender**: Female = 73.3% Male = 25.2% 1.5% did not indicate gender • **Age Ø (range):** Median = 16.40, ± 2.14 (20–65) • **Years of experience Ø (range)**: NA	Depression, Anxiety, Stress (via DASS-21)	Fear of COVID-19 (via Fear of COVID-19 Scale)	Resilience (via Brief Resilience Scale), Avoidance as possible resource (via Experiential Avoidance Scale)	Low
**Serrão et al.**	2020	Portugal	cross-sectional	**HCW (n = 2008)** • allied health professionals from dentistry, nursing, medicine, and pharmacy (n = 707, 35.2%) • physicians (n = 511, 25.4%) • nurses (n = 409, 20.4%), • pharmacists (n = 88, 4.4%) • psychologists (n = 83, 4.1%) • nutritionists (n = 72, 3.6%) • healthcare assistants (n = 29, 1.4%) • workers in allied areas (n = 21, 1%)	• **Gender**: Female = 1678, 83.6%) Male = 330 (16.4%) • **Age Ø (range):** 38 ± 10 (NA) • **Years of experience Ø (range):** NA (<5 - > 15)	Burnout (via CBI)	Depression (via DASS-21), COVID-19 related stressors such as frontline working position, COVID-19 tested, and direct contact with infected people (via self-constructed items)	Psychological Resilience (via RS)	Moderate
**Shahrour & Dardas**	2020	Jordan	cross-sectional	**Nurses (n = 448)**	• **Gender**: Female = 328 (73%) Male = 120 (27%) • **Age Ø (range):** 32.0 (20–58) • **Years of experience:** 10 ± 7 (1–33)	Psychological distress (somatization, depression and anxiety via BSI-18)	Acute Stress Reaction (via SASRQ)	Self-Efficacy (via Trauma Coping Self-Efficacy Scale)	Moderate
**Sharma et al.**	2020	India	cross-sectional	**HCW (n = 184)** **•** nursing staff (n = 77, 41.8%) **•** doctors (n = 72, 39.1%) **•** others (n = 35, 19%)	• **Gender**: Female = 108 (58.70%) Male = 76 (41.30%) • **Age Ø (range):** NA (21 - >50) • **Years of experience Ø (range):** NA	Depression, Anxiety, Stress (via DASS-21), Insomnia (via ISI)	Multiple COVID-19 related stressors including fear of infection, transmission stigma, workplace pressure, etc (self-constructed item)	Coping (via Brief COPE)	Moderate
**Si et al.**	2020	China	cross-sectional	**HCW (n = 863)** **•** Doctors (n = 377, 43.7%) **•** Nurses (n = 211, 24.4%) **•** other health workers (n = 275, 31.9%)	• **Gender**: Female = 610 (70.7%) Male = 253 (29.3%) • **Age Ø (range):** NA (<29 - >50) • **Years of experience Ø (range):** NA	Depression, Anxiety, Stress (via DASS), PTSD intrusion & arousal (via IES-6)	Perceived threat by COVID-19 (self-constructed questionnaire), Stigmatization, High-risk job, fear of infection	Coping (via SCSQ), perceived social support (via PSSS)	Moderate
**Soto-Rubio, Giménez-Espert, Prado-Gascó**	2020	Spain	cross-sectional	**Nurses (n = 125)**	• **Gender**: Female = 79.1% Male = 20,9% • **Age Ø (range):** 43.37 ± 11.58 (24–63) • **Years of experience Ø (range):** NA	Burnout (via CESQT)	Psychosocial risk (workload, lack of organizational justice, role conflict, interpersonal conflicts, psychosomatic health problems [via UNIPISCO]), emotional work (FEWS)	Emotional intelligence (via TMMS-24), social support and job satisfaction (UNIPISCO)	High
**Tahara et al.**	2020	Japan	cross-sectional	**HCW (n = 661)** **•** physician 8 (1,2%) **•** nurse 8 (1,2%) **•** physical therapist 122 (18,5%) **•** occupational therapist 507 (76,7%) **•** speech therapist 16 (2,4%)	• **Gender**: Female = 354 (53.6%) Male = 307 (46.4) • **Age Ø (range):** NA (21- >40) • **Years of experience Ø (range):** NA (1->25)	Mental health status (via GHQ-12)	general health condition and anxiety over COVID-19 (visual analog scale)	"Satisfaction with leisure, satisfaction with job, satisfaction with daily life activities, and satisfaction with new activities started since social distancing began (via self-constructed items based on COMP), Coping strategies (via open ended questions and not included in logistic regression analysis)"	Moderate
**Tran et al.**	2020	Vietnam	cross-sectional	**HCW (n = 7124)** **•** 49.3% nurses, **•** 28.8% doctors, **•** 21.9% were other HCW	• **Gender**: Female = 66,2% Male = 33.8% • **Age Ø (range):** 34.4 ± 8.8 (21–60) • **Years of experience Ø (range):** NA	Depression (via PHQ-9), Quality of life (HRQoL), Anxiety (via GAD-7)	suspected health problems similar to symptoms of COVID-19 (S-COVID-19-S)	Health literacy (via 12-item questionnaire), Health-related behaviors (self-constructed items)	Moderate
**Vagni et al.**	2020, Oct 31	Italy	cross-sectional	**Red Cross volunteers (n = 494)** **•** Group A “Health” (n = 186, 37.7%) **•** Group B “Social” (n = 151, 30.6%) **•** Group C “Emergency” (n = 157, 31.7%)"	• **Gender**: Female = 280 (56.7%) Male = 214 (43,3%) • **Age Ø (range):** Female: 44.40 ± 12.92 (18–75) Male: 47.25 ± 13.5 (18–75) • **Years of experience Ø (range):** NA	Burnout (via MBI–HSS)	Stress (via ESQ, self-constructed original stressor questionnaire)	Resilience (via DRS-15)	Moderate
**Vagni et al.**	2020, Sept	Italy	cross-sectional	**Emergency workers (n = 513)**	• **Gender**: Female = 286 (55,75%) Male = 220 (44,25%) • **Age Ø (range):** Male: 47.10 (17–65) Female: 44.49 (16–65) • **Years of experience Ø (range):** NA	Avoidance, Arousal, Intrusion (secondary trauma; via STSS-I)	Emergency stress (via ESQ incl. Items on COVID-19)	Resilience (via DRS-15), Coping (via CSES-SF)	Moderate
**Woon et al.**	2020	Malaysia	cross-sectional	**HCW (n = 399)**	**•** Gender: Female = 292 (73.2%) Male = 107 (26.8%) **•** Age Ø (range): NA (18–60) **•** Years of experience Ø (range): NA	Depression, Anxiety (via DASS-21)	Different self-constructed questions associated with COVID-19 such as "Were you afraid of being frequently exposed to COVID-19 patients?"	Social support (via MSPSS), various personal factors (via self-reported questionnaire)	Moderate
**Xiao et al.**	2020	China	cross-sectional	**HCW (n = 180)** **•** Doctors (n = 82) **•** Nurses (n = 98)	**• Gender**: Female = 129 (71,7%) Male = 51 (28,3%) **• Age Ø (range):** 32.31 ± 4.88 **• Years of experience Ø (range):** NA (<2 - >5)	Anxiety (via SAS), sleep quality (PSQI)	Acute stress (via SASR)	social support (SSRS), self-efficacy (GSES),	Low
**Yildirim et al.**	2020	Turkey	cross-sectional	**HCW (n = 204)** **•** Doctors (47.55%) **•** Nurses (22,06%) **•** other care workers such as medical assistants (30,39%)	**• Gender:** Female = 102 (50%) Male = 102 (50%) **• Age Ø (range):** 32.92 ± 7.01 **• Years of experience Ø (range):** NA	Depression, Anxiety, Stress (via DASS-21)	Coronavirus fear (3 self-constructed items), perceived risk to be infected (2 self-constructed items)	Resilience (via BRS)	Low
**Young et al.**	2020	USA	cross-sectional	**HCW (n = 1685)** **•** Resident or fellow (n = 76) **•** Student or trainee (n = 23) **•** Clinical or medical staff (n = 1290) **•** Administration (n = 110) **•** Retired, returned for covid (n = 9) **•** Did not identify (n = 177)	**• Gender**: Female = 1096 (76%) Male = 353 (24%) not identified = 236 **• Age Ø (range)**: 33% (464 of 1,399) of the sample were at least age 60, NA **• Years of experience Ø (range):** NA	Depression (via PHQ-9), Anxiety (via GAD-7)	perceived risk of getting infected with COVID-19 or experiencing complications, any preexisting health conditions,	perception of ability to say no to work demands	Moderate
**Yörük, Güler**	2020	Turkey	cross-sectional	**HCW (n = 377)** **•** Midwives (n = 204) **•** Nurses (n = 173)	**• Gender**: Female = 100% Male = 0% **• Age Ø (range):** 32.20 ± 8.11 (20–54 years) **• Years of experience Ø (range):** 9.74 ± 8.55 (1–32 years)	Depression (via BDI)	Perceived Stress (via PSS), Burnout (via MBI), anxiety about COVID-19 infection of self and family, caring for COVID-19 patients, weekly working hours	Resilience (via RSA)	Low

### Prevalence of increased mental health problems in HCWs during the COVID-19 pandemic

Eight studies included in this review suggest higher levels of mental health problems in HCWs compared to the average in the general population [26–33]. Due to the heterogeneity of measures a specific range could not be identified. Four studies report on elevated levels of mental health problems compared to pre-pandemic scores in HCWs [34–37]. Three reports found mental health of HCWs to be worse during the COVID-19 pandemic compared to data gathered from other pandemic or disastrous events [38–40]. One study found measures of anxiety and depression not to reach the clinically relevant cut-off point [41]. The remaining thirty reports did not provide a statement on how their data on mental health issues in HCWs compares to other cohorts.

### The interplay of various mental health constructs, psychosocial resources, and pandemic burden

#### General mental health constructs

Eleven reports on general mental health constructs were included in the review. Even though most authors of those studies subsumed similar constructs such as measures of depression, anxiety, somatization, and stress within their general mental health constructs, they chose a wide variety of names such as “mental health well-being”, “psychological distress”, “mental health problems” and other. Therefore, in the following section these terms will be used interchangeably. Ten of these studies applied a cross-sectional design while one study group generated longitudinal data. In terms of statistical analysis most studies used regression models (seven) to analyze their data. Three studies applied structural equation modeling and one report used a multilevel modeling approach.

Three cross-sectional studies examined the associations between general mental health constructs, **resilience, coping** and pandemic stressors. Bettinsoli et al. [26] surveyed 580 HCWs (40% female) and applied hierarchical linear regression analysis to their data. With respect to the focal variables of the study they found both resilience and self-efficacy coping to be statistically significant protective factors while separation distress and emotional symptoms appeared to be risk factors for mental health problems. The indirect effect between direct exposure to COVID-19 and mental health problems was significantly explained by emotional symptoms and, to a lesser extent, self-efficacy. At this point it must be mentioned that the terms “protective factor” and “risk factor” do not imply a causal but merely statistical relationship and the reader shall keep this in mind when reviewing the following presentation of study results.

Two studies [42, 43] used structural equation modelling to examine the mediating role of resilience and coping with respect to the positive association between pandemic burden and mental health problems. In their survey on 421 Nurses (93.6% female) Lorente et al. [42] found that–in contrary to what was expected–problem-focused coping was positively and emotion-focused coping as well as resilience were negatively associated with psychological distress. All pandemic stressors (work overload, insufficient preparation, lack of support, death and fear of infection) were significantly and positively related to mental health problems. Problem-focused coping partially mediated the relationship of work overload (ß = 0.23), fear of infection (ß = 0.34), and insufficient preparation to deal with work demands (ß = −0.38) with psychological distress. Emotion-focused coping partially mediated the association between fear of infection (ß = −0.34) and psychological distress. The authors further examined the mediating role of resilience with respect to the association between coping style and mental health problems and found that resilience mediated the effect of emotion-focused but not problem-focused coping on psychological distress.

Secer et al. [43] examined to role of resilience and experiential avoidance with respect to mental health problems (termed as “low psychosocial adjustment skills”) and the fear of COVID-19 in 390 HCWs (73.3% female) applying structural equation modeling. Results of the final SEM-model revealed that the impact of fear of COVID-19 on mental health problems was indirectly predicted by experiential avoidance (positively) and psychological resilience (negatively).

One cross-sectional survey on 725 physicians (72% female) examined the role of **resilience**, **coping strategies** and **psychological flexibility** with respect to psychological distress and pandemic stressors, such as COVID-19 anxiety and pandemic concerns [44]. Hierarchical regression analysis revealed that in terms of psychosocial resources psychological flexibility, resilience, and “knowing I did all that I could” (coping strategy) were significant protective factors for psychological distress. Using sedatives as a coping strategy turned out to be a significant risk factor for mental health problems as were COVID-19 social concerns (family, partner, and friends) and COVID-19 anxiety (health concerns). Economic, civil and health system concerns regarding COVID-19 did not reach statistical significance in the model. The same goes for a variety of coping strategies such as physical and sexual activity, reading books, alcohol, nicotine, and drug use, working, as well as humor.

Two cross-sectional studies focused on **social support** and **coping strategies** as psychosocial resources with regard to pandemic stressors and psychosocial distress in nurses [31, 38]. In their survey on 1364 nurses (79% female) Liu et al. [38] were able to show that participants who lived alone, had closer first-line contact with COVID-19 infected patients, and had higher social support scores displayed lower incidence of mild-to-moderate distress in univariate logistic regression analysis. However, when they conducted multivariable regression analysis only higher social support scores remained significant. Coping strategies did not reach statistical significance.

Nie et al. [31] surveyed 263 nurses (76.7% female) and found that perceived social support was a significant protective factor while negative coping was identified as a risk factor for psychological distress in multiple logistic regression analysis. Further, working in an Emergency Department, concern for family, being treated differently because of working in hospital, and COVID-19 related stress symptoms were positively associated with mental health problems while effective precaution measures were found to be an additional protective factor.

One cross-sectional study on 4618 HCWs (86.7% female) [45] also included measures of **social support** (“good family relationships”) and coping strategies but did not include coping as a predictor in their logistic regression model on psychological distress. They found that good family relationships were a significant protective factor for psychological distress while at the same time the perceived risk of contracting the virus as well as having a COVID-19 acquaintance significantly raised the risk for psychological distress. Furthermore, the study identified “having a good feeling about one’s health condition” as an additional protective factor, which seemed to partially mediate the relationship between profession and the risk for mental health problems. The level of exposure to COVID-19 did not reach statistical significance in the model.

Britt et al. [46] conducted a longitudinal study on 97 emergency medicine workers (39.2% female) to examine factors associated with fluctuations in mental health strain during the COVID-19 pandemic. In terms of psychosocial resources, they focused on measures of **social support** and **meaningful work** both of which were not found to be predictive of mental health strain over the course of six weeks in their multilevel modeling approach. Those psychosocial resources did also not interact with COVID-19 demands to predict mental health strain. On the other hand, workload (hours worked the prior week) interacted significantly with COVID-19 personal demands (e.g., “fear of getting sick and/or dying myself”), but not with COVID-19 work demands (e.g., “shortage of personal protective equipment”) to predict mental health strain in emergency medicine personnel.

One cross-sectional study [47] on 448 nurses (73% female) exclusively focused on **self-efficacy coping** as the major psychosocial resource. The authors found that self-efficacy coping was a significant protective factor for psychological distress in their regression model while more acute stress significantly magnified the risk for psychological distress.

Another cross-sectional study by Krok & Zarzycka [48] on 226 HCWs (58.8% female) of various professions examined the relationship of risk perception of COVID-19, **meaning based resources**, **coping** and psychological well-being. By using structural equation modeling they were able to show that there was a significant direct negative effect of risk perception of COVID-19 on psychological well-being. Further, problem-focused and meaning-focused coping accounted for indirect effects of COVID-19 risk perception on psychological well-being indicating that higher levels of COVID-19 risk perception were related to a more frequent use of problem- and meaning-focused coping strategies which in turn were associated with higher levels of psychological well-being. Additionally, a similar indirect effect was found for the association between meaning-based resources and psychological well-being, which was also mediated by a more frequent use of problem- and meaning-focused coping strategies.

Finally, one cross-sectional study on 661 HCWs (53.6% female) [33] focused on some **behavioral measures** of psychosocial resources such as “**satisfaction with leisure”, “satisfaction with daily life activities”, and “satisfaction with new activities started since social distancing began”** and their association with mental health status. They found that only high satisfaction with new activities started since social distancing began was a significant protective factor for poor mental health. The results also show that “less communication than usual with friends” and “high anxiety over COVID-19” were pandemic stressors which significantly raised the odds for poor mental health status in HCWs.

#### Anxiety

We identified 18 cross-sectional studies that included standardized measures of anxiety and met our inclusion criteria.

Five reports [27, 29, 40, 49, 50] investigated the association of **resilience** and pandemic burden with anxiety. Four of these reports found high scores of psychological resilience to be a significant protective factor for symptoms of anxiety displaying mostly moderate effect sizes [27, 29, 40, 50]. With regard to pandemic burden the studies identified the concern about the potential infection of others such as family members [29, 40], anxiety about being infected [40, 50], mental or emotional exhaustion [29, 40], a high susceptibility to emotions and behaviors of other people [27], depersonalization [29], measures of workload [29], feeling obligated to go to work [40], and lack of knowledge about prevention and protection [40] as significant risk factors for elevated levels of anxiety. In their mediation analysis Yildirim et al. [50] found that the significant positive association of coronavirus fear and anxiety was partially mediated by elevated levels of resilience. The perceived risk of being infected with the virus was not related to measures of anxiety.

Pang et al. [51] did not only include **resilience** but also **coping styles** as a measurement of psychosocial resources in their study on 282 nurses (88.7% female). They found that resilience as well as a positive coping style were significant protective factors for anxiety with moderate effect sizes. Negative coping style and low sleep quality were positively associated with higher levels of anxiety in their model. Pandemic burden such as workload did not reach statistical significance.

Kim et al. [34] were able to show that high **resilience** and **social support** as measured by high family functioning were significant negative predictors while caring for COVID-19 patients was a significant risk factor for moderate/severe levels of anxiety in 320 nurses (94.4% female). **High spirituality** on the other hand did not reach statistical significance.

Four studies investigated the relationship between measures of **coping**, pandemic burden and anxiety [30, 41, 52, 53]. All reports identified significant associations between measures of coping and anxiety levels in HCWs. Positive religious coping [41], self-efficacy coping [30], and approach (vs. avoidant) coping [53] were found to be protective factors while conversely negative vs. positive coping [52] and negative religious coping [41] were positively associated with symptoms of anxiety. Most prominent risk factors with regard to pandemic burden were increased workload [30, 52], (work) stress [30, 53], respiratory/digestive tract symptoms in the past two weeks [52], and perceiving multiple stressors [53].

We identified two reports that included measures of **coping** and **social support** as potential psychosocial resources to mitigate the effect of pandemic burden on anxiety [32, 54]. Si et al. [32] identified active coping and perceived social support as significant protective factors for symptoms of anxiety in their regression model, even though the effect size of the negative association between perceived social support and anxiety was very small (β = –0.072, p < 0.001). Confirmed COVID-19 cases in the living community, stigmatization/distancing, working in a high-risk job and passive coping were found to be significant pandemic risk factors for anxiety. In their study on 180 HCWs (71.7% female) Xiao et al. [54] used structural equation modelling and found that social support negatively affected anxiety and acute stress scores while there was a positive association of social support and self-efficacy scores. Anxiety significantly affected levels of stress and reduced self-efficacy and sleep quality.

Three studies reported on the associations of **social support**, pandemic burden and levels of anxiety in HCWs [35, 55, 56]. Only one study by Ni et al. [55] was able to identify overall higher (vs. lower) social support scores to be negatively associated with anxiety. Nevertheless, Woon et al. [56] found a subscale of their support measure (higher perceived social support from friends) to be the only significant protective factor for anxiety while the family and significant other support scores were not. In terms of pandemic burden the fear from frequent exposure to COVID-19 patients and not knowing whether the area of living was highly prevalent for COVID-19-positive cases were identified as significant risk factors for symptoms of anxiety [56]. Confirmed close contact with COVID-19, living in a neighborhood with COVID-19 cases, and time spent on COVID-19 news did not reach statistical significance [55].

One study on 7124 HCWs examined the association of **health literacy, health-related behaviors**, and pandemic burden with symptoms of anxiety [57]. The authors found unchanged or healthier diet, unchanged or more physical exercise, and higher scores in health literacy to be significant protective factors while unchanged or more smoking and unchanged or more drinking alcohol were identified as risk factors for elevated levels of anxiety. The most prominent risk factors in terms of pandemic burden were involvement in COVID-19 response and working in a frontline facility.

Finally, one report on 1685 HCWs (76% female) [58] examined the **“ability to say no to work”,** which we interpreted as a measure of psychosocial resources (i.e. self-care) for the purpose of this review, in respect to symptoms of anxiety. Results indicate that the ability to say no to work was a statistically significant protective factor for symptoms of anxiety. Furthermore, a very high perceived risk of contracting coronavirus, endorsed barriers to working, and being away from home for at least 1 week were found to positively associated with symptoms of anxiety.

#### Depression

Sixteen cross-sectional studies used measures of symptoms of depression as a final outcome variable. Fifteen of these studies applied regression analysis to identify statistically significant predictors of depressiveness, while one study [50] used mediation analysis in order to elucidate relations between symptoms of depression, psychosocial resources and pandemic burden.

Four cross-sectional studies exclusively focused on **resilience** as a psychosocial resource for HCWs [29, 49, 50, 59]. All four of these reports found resilience to be a statistically significant protective factor for symptoms of depression. Three reports included the level of perceived exposure to COVID-19 infected patients or colleagues such as “providing care for confirmed or suspected cases of COVID-19” or “At work, being with people who might have COVID-19” [29, 49, 59], which none of them found to be a significant pandemic risk factor in their regression models including resilience. However, one study [29] was able to show that HCWs who thought that it is very likely that they will be infected with COVID-19 were more likely to display symptoms of depression even though the effect was very small (ß = 0.056, p < 0.05). The same goes for their items “being very concerned that someone with whom you live may be infected” (ß = 0.064, p < 0.01) and “concern over possible infection of a family member you do not live with” (ß = 0.060, p < 0.01) [29]. Another study [59] found no significant association between the fear of infection of self or family members and depressive symptoms. Working in an isolation ward was found to be a significant risk factor for symptoms of depression although it is not clear which pandemic stressors specific to the isolation ward contributed to the effect [49]. Two of the surveys [29, 59] included measures of workload such as weekly working hours or the number of on-call hours per month but only Luceño-Moreno et al. [29] were able to identify workload as a significant risk factor for symptoms of depression, again reaching a very small effect size in the overall model (ß = 0.042, p < 0.05). When examining the association of burnout subscales such as emotional exhaustion, depersonalization and personal accomplishment with symptoms of depression two surveys [29, 59] identified emotional exhaustion as a significant risk factor, while only one [29] was able to show the same for depersonalization. High personal accomplishment was found to be an additional protective factor for symptoms of depression in one study [29] while reduced personal accomplishment did not reach statistical significance in the other [59].

One cross-sectional survey on 204 HCWs (50% female) used mediation analysis in order to identify the associations between the perceived risk of contracting coronavirus, coronavirus fear, depression, anxiety, stress and **resilience** [50]. The results revealed that perceived risk of contracting coronavirus significantly predicted coronavirus fear (β = 0.54, p < 0.001) but was a non-significant predictor of resilience. Coronavirus fear fully mediated the effect of perceived risk on resilience (β = −.32, p = < 0.001). Perceived risk was a significant predictor for symptoms of depression (β = 0.21, p = < 0.001) while this relationship was mediated by coronavirus fear. The effect of coronavirus fear on symptoms of depression on the other hand was significantly mitigated by resilience.

In their cross-sectional survey on 320 nurses (94.4% female) Kim et al. [34] did not only include measures of **resilience** but also **social support** (high family functioning) and **spirituality** in terms of psychosocial resources. Concerning symptoms of depression, they found that only high spirituality (OR = 0.38 [0.21–0.66], p < 0.001) and high family functioning (OR = 0.40 [0.23–0.69], p < 0.001) but not resilience were statistically significant protective factors in their multivariate logistic regression. Being quarantined or self-isolated (OR = 2.68; [1.55–4.63], p < 0.001) was a positive predictor for symptoms of depression, while being involved in COVID-19 patient care was not.

One cross-sectional study by Pang et al. [51] on 282 nurses (88.7% female) examined the associations between **coping style, resilience,** sleep quality and duration, daily working time and participation in EBOLA and SARS rescue in respect to symptoms of depression. They identified resilience (ß = –0.239, p < 0.001), positive coping style (ß = –0.222, p < 0.001), negative coping style (ß = 0.328, p < 0.001), and low sleep quality (ß = 0.152, p = 0.003) as explanatory factors for symptoms of depression while participation in Ebola and SARS rescue, daily working time, and daily sleep duration did not reach statistical significance in their regression model.

Three cross-sectional surveys [35, 55, 56] used measures of **social support** as a variable for psychosocial resources and examined the association with symptoms of depression in HCWs. Only one study [55] found social support to be a protective factor for symptoms of depression in HCWs, while in the other two surveys [35, 56] social support did not reach statistical significance. Both studies which found measures of social support not to be a statistically significant protective factor for symptoms of depression reported measures for fear of being infected with COVID-19 as risk factors in their regression models [35, 56]. Furthermore, Woon et al. [56] were able to show a statistically significant positive association between symptoms of depression and high prevalence rates for COVID-19 in area of living while [35] reported the same for measures of workload (number of days worked all night), “inadequate level of knowledge of COVID-19 prevention” and HCWs perceived troubles at work. The perceived ability to maintain current intensity of work for more than one month was found to be an additional statistical significant protective factor in terms of probable depression [35].

Four cross-sectional studies [28, 41, 52, 53] examined the association between various **coping styles** and symptoms of depression. Chen et al. [52] reported a mostly negative coping style to be the most prominent risk factor for symptoms of depression in their step-by-step multiple logistic regression analysis among further variables such as an increase in workload, respiratory or digestive tract symptoms in the past two weeks, specific tests related to COVID-19, and symptoms of burnout in 902 HCWs (68.6% female). Working in front-line (vs. second line) did not reach statistical significance in their model. Chow et al. [41] focused on positive and negative religious coping with respect to symptoms of depression in 200 HCWs (60.5% female) and found positive religious coping to be a statistically significant protective factor for probable depression while negative religious coping was a risk factor. Effect sizes were very small (ß = -0.019, p = 0.025 and ß = 0.052, p < 0.001). Krammer et al. [28] used their first measurement of a longitudinal survey for cross-sectional analysis in 100 HCWs (74.4% female). They applied hierarchical regression models in order to find statistically significant predictors for symptoms of depression while also including coping strategies such as “positive thinking”. In terms of pandemic burden and psychosocial resources they only found general distress to be a significant predictor for symptoms of depression. Neither positive thinking nor traumatic experiences, fear of infection, fear of infecting family, fear of contact, work stress, and alcohol- and nicotine consumption reached statistical significance. Nevertheless, in studies with small sample sizes and limited statistical power statistical predictors might not reach statistical significance even though they are clinically relevant. In this case fear of infection and fear of contact overall showed beta-coefficients of -0.35 and 0.29 which might point to the clinical relevance of these variables. Lastly, Sharma et al. [53] were able to show that in their cross-sectional analysis on 184 HCWs (58% female) approach (vs. avoidant) was a significant risk factor for symptoms of depression even though the effect was very small (aOR: 1.070 [1.010–1.134], p = 0.021). Anxiety and stress were found to be further risk factors while profession and number of (pandemic) stressors did not reach statistical significance in their model.

One cross-sectional study by [32] on 863 HCWs (70.7% female) included measures of **perceived social support and coping styles** as psychosocial resources. They reported significant protective effects of active coping and perceived social support for symptoms of depression even though the effect size of social support was very small (β = –0.064, p < 0.001). Passive coping, confirmed cases of COVID-19 among relatives and friends, stigmatization/distancing, and working in a high-risk job were found to be significant risk factors for probable depression in their study. Being quarantined or isolated, alcohol consumption, confirmed cases in the living community, and fears of infection did not reach statistical significance in the model.

Another cross-sectional survey on 7124 HCWs (66.2% female) [57] examined the association of **health literacy and health-related behaviors** such as physical activity, diet, smoking and alcohol consumption with symptoms of depression. They found that an unchanged or healthier diet, unchanged or more physical exercise and increments in health literacy were protective factors for symptoms of depression in their multivariable regression model. On the other hand, working in a frontline facility, being involved in COVID-19 response, unchanged or more smoking and unchanged or more drinking alcohol were identified as risk factors for probable depression. Having experienced COVID-19 like symptoms did not reach statistical significance. Interaction analysis revealed that all interactions between COVID-19 response involvement and health literacy/health behavior variables except “being involved x unchanged/healthier diet”were significant in predicting probable depression.

Finally, one cross-sectional study on 1685 HCWs (76% female) [58] examined the **“ability to say no to work”**, which we interpreted as a measure of psychosocial resources (i.e. self-care) for the purpose of this review, in respect to symptoms of depression. It was found to be a significant protective factor while the very high perceived risk for contracting coronavirus, endorsed barriers to working, and being away from home for at least 1 week were stressors related to pandemic burden that showed a positive association with probable depression.

#### Burnout

Eight cross-sectional studies included in this review investigated the relationship between various psychosocial resources, burnout and pandemic burden. All reports used regression models to identify risk factors of burnout while three studies additionally applied mediation/moderation analysis.

Two studies [37, 60] focused on measures of **resilience** as a psychosocial resource and applied hierarchical regression analysis to elucidate relations with burnout and pandemic stressors. Both studies found resilience to be a significant protective factor for burnout. Serrao et al. [37] surveyed 2008 HCWs (83.6% female) using the Copenhagen Burnout Inventory (CBI) and constructed hierarchical regression models for each subscale (personal, work-related and client-related burnout). Results show that resilience was a significant protective factor for personal, work-related and client-related burnout and played a partially mediating role in the significant association between depression and all burnout subscales since the absolute value of the depression’s standardized regression coefficient (ß) reduced from 0.530 to 0.480, 0.522 to 0.476, and 0.352 to 0.305 after inclusion of resilience in the model. In terms of pandemic burden frontline working position, having a diagnosed health problem, and having direct contact with infected people remained statistically independent risk factors for personal and work-related burnout. For client-related burnout “direct contact with infected people” and “death of a relative or friend during the pandemic period” remained significant risk factors in the final model of hierarchical regression.

Vagni et al. [60] investigated burnout in 494 Red Cross volunteers (56.7% female) via the Maslach Burnout Inventory Human Service Survey (MBI-HSS) and found measures of resilience (“hardiness”) to be a significant protective factor for all three subscales of burnout (emotional exhaustion, depersonalization and personal accomplishment). There was a quite significant difference between model 2 and model 3, when stressors were included, in terms of the predictive power of hardiness for emotional exhaustion (model 2: ß = –0.277, p < 0.001 vs. model 3: ß = –0.087, p < 0.05) and depersonalization (model 2: ß = –0.215, p < 0.001 vs. model 3: ß = –0.104, p < 0.05) even though remaining significant. In terms of pandemic burden physical, emotional, and cognitive stress were identified as significant risk factors for emotional exhaustion. Organizational-relational and cognitive stress were statistically significant positive predictors for depersonalization while caring for COVID-19 patients, inefficacy-decisional, emotional, and cognitive stress were associated with reduced personal accomplishment in the study population. The specific measure of COVID-19 stress and weekly working hours were not identified as risk factors in any of the regression models. Caring for COVID-19 patients although displaying predictive power for reduced personal accomplishment was neither associated with emotional exhaustion nor depersonalization.

We identified three reports [61–63] that included **social support** as a psychosocial resources and investigated the associations with burnout while accounting for measures of pandemic burden. Two studies found their measures of social support to be significantly associated with symptoms of burnout [61, 62] while one did not [63]. Manzano Garcia et al. [62] surveyed 771 nurses and found social support to be a significant protective factor for burnout in their hierarchical regression models predicting higher levels of burnout. In terms of pandemic burden, they identified work overload and perceived threat of COVID-19 to be significant risk factors. Ultimately, role conflict, role ambiguity, and autonomy did not reach statistical significance even though role conflict and autonomy were significant before fear of COVID-19 was added to the model. Further analysis revealed that there was a significant interaction between social support and perceived threat of COVID-19 reducing the protective effect of social support on burnout significantly (from ß = −0.153, p < 0.001 to ß = −0.110, p < 0.001).

Roslan et al. [61] used a mixed-method approach (online questionnaire and qualitative interviews) to investigate the relationship between social support, spiritual routines, pandemic burden and burnout in 933 HCWs. For the purpose of this review only results from the statistical cross-sectional analysis are reported. The authors used multiple logistic regression analysis to identify risk factors for all three subscales of the CBI (personal, work-related, and patient-related burnout). Their results reveal that the perceived inadequate psychosocial support at work was a significant risk factor with respect to all three subscales of burnout. Furthermore, irregular spiritual routines were found to be positively associated with work-related burnout. In terms of pandemic burden all three subscales of burnout were positively associated with the direct involvement with COVID-19 patients and suffering from some kind of medical illness (pre-existing medical condition). Additionally, working more than 60 hours per week was found to be a significant risk factor for personal and work-related but not patient-related burnout.

Soto-Rubio et al. [63] surveyed 125 nurses (79.1% female) and did not find a significant association of social support with burnout in their hierarchical regression models. Nevertheless, they found emotional repair as a measurement of emotional intelligence to be a significant protective factor. Additionally, emotional work, interpersonal conflict and role conflict were identified as risk factors for symptoms of burnout.

One cross-sectional study focused on the level of **optimism** about overcoming COVID-19 as a psychological resource in 169 HCWs (58.6% female) and investigated the associations of optimism with job stress, caused by COVID-19, and emotional exhaustion as a measure of burnout [64]. They were able to show that higher levels of optimism about overcoming COVID-19 were negatively associated with job stress and emotional exhaustion.

One cross-sectional study [65] explored **self-efficacy** as a potential protective factor for burnout considering pandemic stressors such as psychological job demand and social stigma in 273 HCWs (50.2% female). The results show that higher scores of self-efficacy were identified as a significant risk factor for compassion fatigue and less compassion satisfaction. In terms of pandemic stressors stigma discrimination, fear of stigmatization and higher psychological job demands were significant risk factors for symptoms of burnout in their model.

Lastly, we identified one cross-sectional study on 497 HCWs (63.4% female) which used measures of cognitive as well as affective **empathy**, **“meaningful work”**, and **professional identification** as psychosocial resources. They conducted separate regression analysis on physicians and nurses to identify risk and protective factors for exhaustion and disengagement as subscales of burnout and were able to show that professional identification was a significant protective factor for exhaustion in physicians but not nurses, as well as for disengagement in both professions. Affective empathy turned out to be a significant risk factor for exhaustion but not disengagement in both professions while meaningful work was identified as protective factor for disengagement only in nurses. Procedural justice was found to be negatively associated with both outcomes and professions. In terms of pandemic burden “task changes due to COVID-19” and “being isolated from family” displayed no significant association with any of the burnout subscales. Nevertheless, a higher workload was associated with a significant increase in exhaustion in both nurses and physicians as well as more disengagement in nurses.

#### Post-traumatic stress

We identified seven studies that included measures of post-traumatic stress symptoms and investigated the relationship with psychosocial resources and pandemic burden in HCWs. Four studies used a cross-sectional design, two generated longitudinal data and one was a predictive cohort study.

Three studies focused on **resilience** as a psychosocial resource of HCWs [29, 39, 66]. Two of the studies found measures of resilience to be a significant protective factor for symptoms of post-traumatic stress [29, 39] even though effect sizes were small. In terms of risk factors in association with post-traumatic stress symptoms these studies identified the level of exposure to COVID-19 (i.e. working in inpatient COVID-19 units) [39], perceived risk of infection (i.e. “thinking that there is a high risk of also becoming infected with COVID-19”) [29], as well as concern about the infection of people HCWs live with [29]. In their hierarchical regression model on longitudinal data Hines et al. [66] did not find any significant predictor and note that their statistical power was limited. Again, taking a look at standardized beta-coefficients supportive work (ß -0.133), social support (ß -0.195) and positive affect (ß -0.111) might potentially be clinically relevant protective factors, while sleep disturbance (ß 0.237) and moral injury baseline score (ß 0.197) could potentially be clinically relevant risk factors in association with post-traumatic stress.

One cross-sectional study on 184 HCWs (50.5% female) including measures of **resilience** and **self-efficacy coping** did not find any significant association of these psychosocial resources with secondary traumatic stress [36]. On the other hand, exposure to patients’ deaths, perceived stress, and emotional exhaustion scores were identified as significant risk factors. COVID-19 specific burdensome factors such as the COVID-19 infection of family members or friends and hours per day spent with COVID-19 patients did not reach statistical significance.

One cross-sectional study on 513 emergency workers (55.8% female) found **resilience** and **emotion-focused coping strategies** (i.e. “stop unpleasant emotions/thoughts”) to be significant protective factors for most symptoms of post-traumatic stress such as arousal, intrusion and avoidance [67]. **Problem-focused coping** did not reach statistical significance in any of their models and effect sizes of emotion-focused coping strategies were very small. COVID-19, emotional and physical stress were identified as significant risk factors for all subscales of traumatic stress. Mediation analysis revealed that 18% of the effect of "total stress" (including cognitive, physical, emotional, organizational-relational, inefficacy decisional, and COVID-19 stress) on arousal and 25% of the effect of "total stress" on avoidance were found to be significantly mediated by resilience, problem focused and emotion focused coping, while the effect of total stress on intrusion was not mediated.

Two studies, one cross-sectional [32] and one longitudinal [68], focused on **coping strategies** and **social support** as psychosocial resources with respect to symptoms of post-traumatic stress and pandemic burden. In their longitudinal study on 221 HCWs (49,8% female) Chew et al. [68] identified problem-solving coping to be a significant protective factor for traumatic stress over time. The use of avoidance coping, levels of perceived stigma and social support were associated with elevated levels of posttraumatic stress. Direction dependence analysis revealed that greater traumatic stress was likely to lead to more social support seeking. Similarly, Si et al. [32] found passive coping next to being highly concerned about COVID-19, stigmatization/distancing, fear of infection, and working in a high-risk job to be associated with elevated symptoms of post-traumatic stress symptoms in 863 HCWs (70.7% female). They did not find any significant association of post-traumatic stress and active coping or perceived social support.

#### Other mental health outcomes

There were five studies that report results on the associations between psychosocial resources, pandemic burden and other mental health outcomes as the ones mentioned above.

One cross-sectional study on 6409 HCWs (72.4% female) examined the relationship between suicidal thoughts and behaviors, other mental health variables such as PTSD, anxiety and depression, pandemic burden and **social support** [69]. The authors were able to show that measures of social support (“living together” and “social network”) were significant protective factors for suicidal thoughts and behaviors. In terms of pandemic burden only being hospitalized because of a COVID-19 infection was found to be a significant risk factor while work related factors such as working overtime or problematic work-life balance did not reach statistical significance. Mental disease variables such as lifetime depression, lifetime anxiety, current clinical depression, and current panic attacks were associated with a higher risk of suicidal thoughts and behaviors. Being hospitalized because of a COVID-19 infection remained by far the strongest predictor in the model.

A longitudinal study on 96 HCWs (51% female) [66] found that moral injury at the three-month follow-up was significantly associated with moral injury/psychological distress and a stressful work environment at baseline. Measures of psychosocial resources such as **resilience** did not reach statistical significance neither did the final model of hierarchical regression since statistical power was very limited.

Krammer et al. [28] conducted hierarchical regression analysis on their cross-sectional data of 100 HCWs to examine the relationship between adjustment disorder, pandemic burden and **coping strategies**. They found general distress and a history of traumatic events to be significantly associated with symptoms of adjustment disorder while measures of coping (positive thinking) and pandemic burden (fear of infection, fear of infecting family, fear of contact, and work stress) did not reach statistical significance. Further variables such as fear of contact (ß 0.14) and positive thinking (ß -0.16) might be of clinical relevance.

Liao et al. [70] investigated the association of various factors with symptoms of acute stress disorder in their cross-sectional study on 1092 clinical nurses (99.5% female). They found all variables of **social support** (friend, family and other) to be significant protective factors while working in an epidemic or non-epidemic department of the hospital vs. going to Wuhan to support in the fight against the pandemic was identified as a significant risk factor for symptoms of acute stress disorder. Measures of self-efficacy did not reach statistical significance in the model.

Finally, one cross-sectional study on 7124 HCWs (66.2% female) aimed to examine statistically significant protective and risk factors for quality of life as a measure of mental health. In terms of psychosocial resources, the authors found that an unchanged or healthier **diet**, unchanged or more **physical exercise**, and higher scores of **health literacy** were associated with higher scores in quality of life while the involvement in COVID-19 response, suspected health problems similar to symptoms of COVID-19, and unchanged or more smoking were identified as risk factors for a low quality of life.

## Discussion

### Aggregation and interpretation of results

Our results as presented above show that resilience is the most consistent statistically significant protective factor in terms of psychosocial resources for general mental health constructs (4 out of 4), anxiety (6 out of 7), depression (5 out of 6), and symptoms of burnout (2 out of 2) in HCWs during the COVID-19 pandemic. Results on the protective effect of resilience for PTSD are mixed (3/5). Overall the notion that resilience may be a crucial element in coping with adverse mental health effects of the pandemic on an individual and societal level [71] is supported. The included studies also identified several pandemic stressors as statistically significant risk factors for mental health: High risk of infection, fear of infection, increased workload, concern about loved ones, loss-associated events (e.g., separation distress or death), inadequate knowledge of infection prevention, being in a risk group (e.g., pre-existing medical condition), and general distress (physical, emotional, cognitive, and organizational). When considering these pandemic stressors, standardized measures of resilience remained a significant protective factor for symptoms of mental health problems in HCWs, which suggests that resilience is a robust psychosocial resource for HCWs in preserving mental health when facing pandemic burden. Nevertheless, the concept of resilience needs to be discussed critically. Resilience as applied in the reports mentioned above lacks a clear and homogeneous definition. Studies used various instruments for measuring resilience such as the Connor-Davidson Resilience Scale (CD-RISC) [34, 39, 40, 51], the Brief Resilience Scale (BRS) [29, 43, 50], the Dispositional Resilience Scale (DRS) [60, 67], the 14-Item Resilience Scale (RS-14) [36], the Brief Resilience Coping Scale (BRCS) [26] and the Resilience Scale (RS) [37], which are each based on different theoretical concepts. The CD-RISC, for instance, measures resilience as a conglomerate of characteristics (traits) such as self-efficacy, sense of humor, patience, optimism, and faith [72]. The BRS conceptualizes resilience rather as a process variable as “the ability to bounce back or recover from stress” [73], which reflects the general debate about the conceptualization of resilience in adult health science [74]. Therefore, despite the consistent results on resilience as a statistical protective factor for mental health this result must be interpreted with caution.

Shedding light on the role of coping styles as a psychosocial resource for HCWs when facing pandemic burden requests to draw a more differentiated picture. Coping strategies are diverse in terms of appraisal (e.g., positive vs. negative) and style (e.g., emotion-focused vs. problem-focused coping). In the studies presented above positive coping as measured by the Simplified Coping Style Questionnaire (SCSQ) [31, 32, 51] and the 20-item Trait Coping Style Questionnaire (TCSQ) [52] mainly refers to active coping such as focusing on a positive aspect (e.g., positive thinking), identifying several different ways to solve problems, or taking situations humorously. Negative coping on the other hand refers to a more passive style of coping such as smoking and alcohol consumption, fantasizing miracles, isolation, or crying alone. Therefore, we will use the more neutral terms of active and passive coping in the following discussion. The results as reported above show that active coping was found to be a mostly consistent protective factor for symptoms of anxiety (3 out of 3) and depression (3 out of 4). Nevertheless, one study found contradicting effects of approach vs. avoidant coping with respect to depressiveness displaying small but significant effect sizes. Passive coping was consistently identified as a risk factor for general mental health constructs (1 out of 1), symptoms of anxiety (3 out of 3), depression (3 out 3), and PTSD (1 out of 1). Furthermore, emotion-focused coping was found to be a protective factor for general mental health problems (2 out of 2) and symptoms of PTSD (1 out of 1). However, evidence on the protective effect of problem-focused coping for mental health is inconsistent. While two studies found problem-focused coping to be a significant protective factor for general mental health problems and PTSD, one survey was not able to confirm the significant association with PTSD and one survey found an adverse effect with respect to general mental health. Possible explanations will be discussed below. Higher levels of self-efficacy coping, a measure of how confident HCWs are in being able to deal with upcoming challenges and stress factors [26], was consistently found to be a protective factor for general mental health problems (2 out of 2) as well as symptoms of anxiety (1 out of 1). Positive religious coping as measured by the Brief Religious Coping Scale (BRCOPE) [41] positive religious coping refers to subscales such as “Looked for a stronger connection with God”, “Asked forgiveness for my sins”, and “Focused on religion to stop worrying about my problems” [75]. Negative religious coping on the other hand includes scales such as “Wondered whether God had abandoned me”, “Felt punished by God for my lack of devotion”, and “Questioned the power of God” [75]. Positive religious coping displayed protective effects for symptoms of anxiety (1 out of 1) and depression (1 out of 1) while negative religious coping was found to potentially exacerbate anxiousness (1 out of 1) and depressiveness (1 out of 1). Lastly, meaning-based coping strategies were found to be a protective factor for general mental health problems (1 out of 1).

In terms of pandemic burden the above-mentioned studies found significant risk factors for mental health problems which may be categorized as follows: Fear of infection, high risk of infection, increased workload, symptoms of physical illness, stigmatization, concern about loved ones, loss-associated events, and general distress. Protective effects of coping as reported above remained robust when considering pandemic burden. Consequently, the notion that an active coping style might be beneficial in reducing symptoms of depression and anxiety while the use of emotion-focused coping strategies tends to protect HCWs from general mental health problems and PTSD when facing pandemic stressors is supported. Passive coping strategies on the other hand seem to add to the exacerbating effect of pandemic burden on mental health.

Finally, social support as a frequently reported psychosocial resource of HCWs was mostly found to be a significant protective factor in terms of general mental health problems (3 out of 4), symptoms of anxiety (5 out of 6) and burnout (2 out of 3) while results on symptoms of depression and PTSD remained inconclusive. Regarding PTSD one study found social support to be a significant risk factor in cross-sectional analysis. Further direction of dependence analysis revealed that HCWs who displayed more symptoms of PTSD were more likely to seek social support. This is a reminder to interpret cross-sectional data with great caution since causal implications are not legitimate and a bidirectional relationship of variables at hand must be considered.

Nevertheless, social support as a potential protective factor for general mental health problems, symptoms of anxiety and burnout remained robust when considering factors of pandemic burden such as: High risk of infection stigmatization, fear of infection, increased workload, being in a risk group, and concern about loved ones.

In sum, our results suggest that resilience, active and emotion-focused coping strategies as well as social support can be considered beneficial when protecting different aspects of mental health in HCWs during the COVID-19 pandemic. The opposite holds true for passive coping strategies and several pandemic stressors faced by HCWs. For that matter high risk and fear of a COVID-19 infection were the most frequently reported sources of pandemic burden. So far it has been shown that measures of psychosocial resources and pandemic burden are simultaneously and significantly associated with mental health outcomes, but specific patterns of interaction between the variables remain unclear.

### The specific interplay of psychosocial resources and pandemic burden

Some studies included in the current review show preliminary evidence for specific patterns within the interplay of pandemic burden and psychosocial resources with mental health variables. Specifically, the negative effect of the exposure to COVID-19 infected patients and the perceived risk of infection on mental health of HCWs seems to be mediated by affect, especially (coronavirus) fear [26, 50]. The subsequent handling of this fear may–at least in part–determine the magnitude of its contradicting effect on mental health. Higher resilience could mitigate the exacerbating effect of coronavirus fear on mental health issues [43, 50]. The same holds true for self-efficacy and emotion-focused coping [26, 42]. On the other hand, the evidence suggests that avoidance and problem-focused coping promotes the positive association between coronavirus fear and mental health problems [42, 43]. In turn, problem-focused coping was shown to be a significant protective factor when it comes to the negative effect of work overload on mental health [42]. To put it in a nutshell, HCWs may need both–emotion- and problem-focused coping strategies–in order to deal with pandemic burden, but both strategies have to be used adequately. Trying to cope with coronavirus fear by using practical problem-solving measures might result in a contradicting effect because the vast majority of HCWs cannot completely avoid triggers of anxiety (i.e., exposure to COVID-19 infected patients) since they work in facilities where COVID-19 cases are potentially treated. Emotion-focused coping strategies should be applied to cope with emotional stress while problem-focused strategies are more effective in reducing burden resulting from rather external and rational stressors such as working conditions (e.g., workload).

With respect to the fear of COVID-19 being a central part of pandemic burden experienced by HCWs, underlying factors such as an anxious/insecure attachment style may expose individuals as especially vulnerable to increased feelings of anxiety, COVID-19 distress and psychological distress symptoms as recent research on the general population suggests [76, 77]. Therefore, HCWs with an insecure/anxious attachment style might be at greater risk of developing mental health problems if no sufficient coping mechanisms are applied. Furthermore, elucidating the central role of emotional reactivity and emotion regulation might contribute to the explanation of a widely reported gender difference regarding mental health problems in HCWs. In their recent systematic review and meta-analyses Kunzler et al. [78] found female gender to be a frequently reported risk factor for increased levels of mental health symptoms in HCWs and the general population during the COVID-19 pandemic which is in line with another systematic literature review by Gilan at al. [79] and several studies included in the current review–even though that was not the question at hand. Recent results from neuroscience suggest that women may tend to be more reactive to negative emotional stimuli as represented by an enhanced activity in the amygdala [80]. When integrating these preliminary results with the evidence presented above one can hypothesize that gender differences regarding mental health during the current pandemic might at least in part be explained by emotional reactivity to pandemic threats (e.g., risk of infection) and their subsequent regulation, leaving female HCWs more prone to mental health problems. This hypothesis will be worth exploring in future research since the vast majority of HCWs identifies as female gender. Obviously, stereotypical societal beliefs about gender roles (e.g., frequent expression of the hero archetype in men) will then have to be added to the equation.

### Quality of evidence

The average study quality was moderate-low. Thirty-three included reports were found to be of moderate quality. Only three studies reached the threshold for high quality while the remaining ten reports were rated as of low quality. A common problem were sampling methods since most studies used convenience samples and were not able to report on characteristics of respondents and non-respondents, which makes the data susceptible to selection bias. For example, one could argue that HCWs that were especially stressed might be less prone to participate in research. 89% of included reports applied a cross-sectional study design, which does not allow for causal interpretation of the data. Nevertheless, several cross-sectional reports presented in this review use phrases that imply a causal relationship between “risk factors” and mental health. Small hints within the limitations do not seem to adequately correct the implicitly drawn picture of causality. Furthermore, most studies that used structural equation modelling or mediation analysis did not report how they controlled for known cofounders such as gender while most regression analysis incorporated such factors in their model. Overall, one explanation for the low study quality might be the nature of the research subject. In times of a global pandemic researchers were forced to act fast and efficient, while also adapting measurement tools to the increased workload of medical personnel to minimize additional burden on study subjects. At the same time the global goal was to identify mental health risks and subsequently deviate staring points for supportive interventions. Nevertheless, the presented results have to be interpreted with caution and need to be considered preliminary evidence that points to important research topics in the future.

### Limitations

First of all, as mentioned above, results of this review have to be interpreted with caution since there are very few longitudinal studies and the overall study quality is rather low. In addition, measurement methods for “mental health”, “pandemic burden” and “psychosocial resources” were varying between reports, which makes it hard to draw stressable conclusions from the data.Secondly, the body of evidence regarding the mental health of HCWs during the COVID-19 pandemic is rapidly growing. Since we conducted the search in February 2021 this review can only cover the pandemic situation as of 2020 and there will surely be newer evidence on the subject that is not included in our paper. Also, by the time of publication pandemic conditions might have been altered by the broad availability of vaccines against the virus, which might affect subjective pandemic burden, psychosocial resources and the mental health of HCWs as a recently published study suggests [81]. Therefore, this review must be declared as a first base of evidence to be extended in the future.

Finally, after extensive systematic literature search, the initial title and abstract screening was performed by one reviewer only. Thus, potentially relevant articles could have been missed. However, two reviewers conducted a fully blinded full-text screening and resolved conflicts accordingly.

### Practical implications (recommendations for interventions) and future research

The results of this review suggest that future interventions aiming to preserve the mental health of HCWs in times of the COVID-19 pandemic should include measures to foster resilience, active coping as well as positive spiritual coping and social support. Since resilience is a heterogenous construct this could mean that interventions may focus on promoting characteristics that are associated with the ability to cope with stress such as identifying personal competence and self-efficacy, the tolerance of negative affect, the positive acceptance of change, relying on secure personal relationships and one’s spiritual resources. With respect to specific coping strategies our results suggest that it might be helpful to distinguish between emotion-focused and problem-focused coping and how to use the corresponding strategies adequately. On a structural level interventions may provide a framework that enables HCWs to sustain preexisting psychosocial resources such as social support through family and colleagues, i.e., by providing free COVID-19 tests to limit the fear of infecting others and by promoting digital communication methods.

Future research exploring metal health factors of HCWs in times of the global COVID-19 pandemic should extend the pool of prospective longitudinal and interventional studies to determine the nature of the various associations between pandemic burden, psychosocial resources, and mental health outcomes as described above and to assess the expected effects of corresponding interventions. Furthermore, research on underlying factors such as emotional reactivity and emotion regulation could be intensified pursuing the goal of identifying specifically vulnerable HCWs beyond the reductionistic perspective of gender (“being female”) as a potential risk factor.

## Conclusion

This paper summarizes evidence on the available literature that explores the specific interplay of psychosocial resources and pandemic burden regarding the mental health of HCWs in times of the 2019 COVID-19 pandemic. We found evidence to support the notion, that HCWs are confronted with several pandemic stressors such as fear of infecting oneself or others, increased workload, concern about loved ones, loss-associated events (e.g., separation distress or death), and general distress. We also found that several psychosocial resources such as resilience, active and emotion-focused coping strategies as well as social support were statistically associated with less mental health problems in HCWs. Regarding coping strategies, we found a possible interaction of stressor type and coping style. Nevertheless, most underlying mechanisms regarding the specific interaction between pandemic burden and the buffering effect of psychosocial resources remain unclear. Prospective longitudinal studies are required to elucidate those missing links.

## Supporting information

S1 Checklist PRISMA checklist. (DOCX)

S1 Appendix Summary of search terms and search strategy. (PDF)

S2 Appendix Full list of relevant instruments used within included studies. (PDF)

## References

[1] RKI—Coronavirus SARS-CoV-2—COVID-19: Fallzahlen in Deutschland und weltweit [Internet]. Robert-Koch-Institut; c2021 [cited 2021 Nov 14]. Available from: https://www.rki.de/DE/Content/InfAZ/N/Neuartiges_Coronavirus/Fallzahlen.html

[2] Epidemiologischer Steckbrief zu SARS-CoV-2 und COVID-19 [Internet]. Robert Koch Institut; c2021 [cited 2021 Nov 14]. Available from: https://www.rki.de/DE/Content/InfAZ/N/Neuartiges_Coronavirus/Steckbrief.html

[3] Bericht zu Virusvarianten von SARS-CoV-2 in Deutschland. Robert Koch Institut; c2021 [cited 2021 Nov 14]. Available from: https://www.rki.de/DE/Content/InfAZ/N/Neuartiges_Coronavirus/DESH/Berichte-VOC-tab.html

[4] Nguyen LH, Drew DA, Graham MS, Joshi AD, Guo C-G, Ma W, et al. Risk of COVID-19 among front-line health-care workers and the general community: a prospective cohort study. Lancet Public Heal. 2020 Sep;5(9): e475–483. doi: 10.1016/S2468-2667(20)30164-X 32745512 PMC7491202

[5] Lucchini A, Iozzo P, Bambi S. Nursing workload in the COVID-19 era. Intensive Crit Care Nurs. 2020 Dec;61: 102929. doi: 10.1016/j.iccn.2020.102929 32893048 PMC7418697

[6] Reper P, Bombart MA, Leonard I, Payen B, Darquennes O, Labrique S. Nursing Activities Score is increased in COVID-19 patients. Intensive Crit Care Nurs. 2020 Oct;60: 102891. doi: 10.1016/j.iccn.2020.102891 32712068 PMC7250773

[7] Chan AOM, Huak CY. Psychological impact of the 2003 severe acute respiratory syndrome outbreak on health care workers in a medium size regional general hospital in Singapore. Occup Med (Lond). 2004 May 1;54(3): 190–196. doi: 10.1093/occmed/kqh027 15133143 PMC7107861

[8] Chong M-Y, Wang W-C, Hsieh W-C, Lee C-Y, Chiu N-M, Yeh W-C, et al. Psychological impact of severe acute respiratory syndrome on health workers in a tertiary hospital. Br J Psychiatry. 2018/01/02. 2004;185(2): 127–133. doi: 10.1192/bjp.185.2.127 15286063

[9] El Gaafary MM, Abd Elaziz KM, Abdel-Rahman AG, Allam MF. Concerns, perceived impacts and preparedness of health care workers in a referral hospital in Egypt in facing influenza (H1N1) epidemic. J Prev Med Hyg. 2010;51(3). 21361114

[10] Matsuishi K, Kawazoe A, Imai H, Ito A, Mouri K, Kitamura N, et al. Psychological impact of the pandemic (H1N1) 2009 on general hospital workers in Kobe. Psychiatry Clin Neurosci. 2012 Jun;66(4): 353–360. doi: 10.1111/j.1440-1819.2012.02336.x 22624741

[11] Raven J, Wurie H, Witter S. Health workers’ experiences of coping with the Ebola epidemic in Sierra Leone’s health system: a qualitative study. BMC Health Serv Res. 2018 Dec 5;18(1): 251. doi: 10.1186/s12913-018-3072-3 29622025 PMC5887191

[12] Talat N, Azam MK, Mirza MB, Singh N, Aziz U, Tahir W, et al. Psychosocial Effects of COVID-19 on Health Care Workers: A Cross Sectional Study from Tertiary Level Pediatric Hospital. Ann King Edward Med Univ. 2020 Jul 11;26(Special Issue SE-): 170–175.

[13] Morawa E, Schug C, Geiser F, Beschoner P, Jerg-Bretzke L, Albus C, et al. Psychosocial burden and working conditions during the COVID-19 pandemic in Germany: The VOICE survey among 3678 health care workers in hospitals. J Psychosom Res. 2021 May;144: 110415. doi: 10.1016/j.jpsychores.2021.110415 33743398 PMC7944879

[14] Schug C, Erim Y, Geiser F, Hiebel N, Beschoner P, Jerg-Bretzke L, et al. [Vaccination willingness against COVID-19 among healthcare workers in Germany: Results from a University Medicine Network survey between November 2020 and January 2021]. Bundesgesundheitsblatt Gesundheitsforschung Gesundheitsschutz. 2021 Sep 23;(April). doi: 10.1007/s00103-021-03418-6 34554277 PMC8458789

[15] Steudte-Schmiedgen S, Stieler L, Erim Y, Morawa E, Geiser F, Beschoner P, et al. Correlates and Predictors of PTSD Symptoms Among Healthcare Workers During the COVID-19 Pandemic: Results of the egePan-VOICE Study. Front psychiatry. 2021;12: 686667. doi: 10.3389/fpsyt.2021.686667 34483985 PMC8416177

[16] Wu P, Fang Y, Guan Z, Fan B, Kong J, Yao Z, et al. The psychological impact of the SARS epidemic on hospital employees in China: exposure, risk perception, and altruistic acceptance of risk. Can J Psychiatry. 2009 May 1;54(5): 302–311. doi: 10.1177/070674370905400504 19497162 PMC3780353

[17] Schug C, Morawa E, Geiser F, Hiebel N, Beschoner P, Jerg-Bretzke L, et al. Social Support and Optimism as Protective Factors for Mental Health among 7765 Healthcare Workers in Germany during the COVID-19 Pandemic: Results of the VOICE Study. Int J Environ Res Public Health. 2021 Apr 6;18(7): 3827. doi: 10.3390/ijerph18073827 33917493 PMC8038794

[18] Schmuck J, Hiebel N, Rabe M, Schneider J, Erim Y, Morawa E, et al. Sense of coherence, social support and religiosity as resources for medical personnel during the COVID-19 pandemic: A web-based survey among 4324 health care workers within the German Network University Medicine. Doering S, editor. PLoS One. 2021 Jul 26;16(7): e0255211. doi: 10.1371/journal.pone.0255211 34310616 PMC8312980

[19] Zhu W, Wei Y, Meng X, Li J. The mediation effects of coping style on the relationship between social support and anxiety in Chinese medical staff during COVID-19. BMC Health Serv Res. 2020 Nov 4;20(1): 1007. doi: 10.1186/s12913-020-05871-6 33148229 PMC7609823

[20] Di Giuseppe M, Nepa G, Prout TA, Albertini F, Marcelli S, Orrù G, et al. Stress, Burnout, and Resilience among Healthcare Workers during the COVID-19 Emergency: The Role of Defense Mechanisms. Int J Environ Res Public Health. 2021 May 14;18(10): 5258. doi: 10.3390/ijerph18105258 34069270 PMC8156145

[21] Garritty C, Gartlehner G, Nussbaumer-Streit B, King VJ, Hamel C, Kamel C, et al. Cochrane Rapid Reviews Methods Group offers evidence-informed guidance to conduct rapid reviews. J Clin Epidemiol. 2021 Feb;130: 13–22. doi: 10.1016/j.jclinepi.2020.10.007 33068715 PMC7557165

[22] Ouzzani M, Hammady H, Fedorowicz Z, Elmagarmid A. Rayyan—a web and mobile app for systematic reviews. Syst Rev. 2016 Dec 5;5(1): 210. doi: 10.1186/s13643-016-0384-4 27919275 PMC5139140

[23] Microsoft Corporation. Microsoft Excel. 2018.

[24] Wells G, Shea B, O’Connell D, Peterson J, Welch V, Losos M, et al. The Newcastle-Ottawa Scale (NOS) for assessing the quality of nonrandomised studies in meta-analyses [Internet]. 2011. Available from: http://www.ohri.ca/programs/clinical_epidemiology/oxford.asp doi: 10.2174/138161211795656729 21492089

[25] Modesti PA, Reboldi G, Cappuccio FP, Agyemang C, Remuzzi G, Rapi S, et al. Panethnic Differences in Blood Pressure in Europe: A Systematic Review and Meta-Analysis. Fuchs FD, editor. PLoS One. 2016 Jan 25;11(1): e0147601. doi: 10.1371/journal.pone.0147601 26808317 PMC4725677

[26] Bettinsoli ML, Di Riso D, Napier JL, Moretti L, Bettinsoli P, Delmedico M, et al. Mental health conditions of italian healthcare professionals during the covid‐19 disease outbreak. Appl Psychol Heal Well-Being. 2020 Oct 5; doi: 10.1111/aphw.12239 33016564 PMC7675316

[27] Huang L, Wang Y, Liu J, Ye P, Chen X, Xu H, et al. Factors Influencing Anxiety of Health Care Workers in the Radiology Department with High Exposure Risk to COVID-19. Med Sci Monit. 2020 Jul 25;26: e926008. doi: 10.12659/MSM.926008 32710536 PMC7401832

[28] Krammer S, Augstburger R, Haeck M, Maercker A. [Adjustment Disorder, Depression, Stress Symptoms, Corona Related Anxieties and Coping Strategies during the Corona Pandemic (COVID-19) in Swiss Medical Staff]. Psychother Psychosom Med Psychol. 2020 Jul;70(7):272–82. doi: 10.1055/a-1192-6608 32688420

[29] Luceño-Moreno L, Talavera-Velasco B, García-Albuerne Y, Martín-García J. Symptoms of Posttraumatic Stress, Anxiety, Depression, Levels of Resilience and Burnout in Spanish Health Personnel during the COVID-19 Pandemic. Int J Environ Res Public Health. 2020 Jul 30;17(15). doi: 10.3390/ijerph17155514 32751624 PMC7432016

[30] Mo Y, Deng L, Zhang L, Lang Q, Pang H, Liao C, et al. Anxiety of nurses to support wuhan in fighting against covid‐19 epidemic and its correlation with work stress and self‐efficacy. J Clin Nurs. 2020 Nov 22; doi: 10.1111/jocn.15549 33141987

[31] Nie A, Su X, Zhang S, Guan W, Li J. Psychological impact of covid‐19 outbreak on frontline nurses: A cross‐sectional survey study. J Clin Nurs. 2020 Aug 26; doi: 10.1111/jocn.15454 32786150 PMC7436701

[32] Si M-Y, Su X-Y, Jiang Y, Wang W-J, Gu X-F, Ma L, et al. Psychological impact of COVID-19 on medical care workers in China. Infect Dis poverty. 2020 Aug 12;9(1): 113. doi: 10.1186/s40249-020-00724-0 32787929 PMC7422468

[33] Tahara M, Mashizume Y, Takahashi K. Coping Mechanisms: Exploring Strategies Utilized by Japanese Healthcare Workers to Reduce Stress and Improve Mental Health during the COVID-19 Pandemic. Int J Environ Res Public Health. 2020 Dec 27;18(1). doi: 10.3390/ijerph18010131 33375444 PMC7795636

[34] Kim SC, Quiban C, Sloan C, Montejano A. Predictors of poor mental health among nurses during COVID-19 pandemic. Nurs OPEN. 2020;00: 1–8. doi: 10.1002/nop2.697 33570266 PMC7753542

[35] Li J, Xu J, Zhou H, You H, Wang X, Li Y, et al. Working conditions and health status of 6,317 front line public health workers across five provinces in China during the COVID-19 epidemic: a cross-sectional study. BMC Public Health. 2021 Jan 9;21(1): 106. doi: 10.1186/s12889-020-10146-0 33422035 PMC7794632

[36] Orrù G, Marzetti F, Conversano C, Vagheggini G, Miccoli M, Ciacchini R, et al. Secondary Traumatic Stress and Burnout in Healthcare Workers during COVID-19 Outbreak. Int J Environ Res Public Health. 2021 Jan 5;18(1). doi: 10.3390/ijerph18010337 33466346 PMC7794988

[37] Serrão C, Duarte I, Castro L, Teixeira A. Burnout and Depression in Portuguese Healthcare Workers during the COVID-19 Pandemic-The Mediating Role of Psychological Resilience. Int J Environ Res Public Health. 2021;18(2). doi: 10.3390/ijerph18020636 33451083 PMC7828555

[38] Liu Y, Long Y, Cheng Y, Guo Q, Yang L, Lin Y, et al. Psychological Impact of the COVID-19 Outbreak on Nurses in China: A Nationwide Survey During the Outbreak. Front PSYCHIATRY. 2020;11. doi: 10.3389/fpsyt.2020.598712 33362609 PMC7759517

[39] Li X, Zhou Y, Xu X. Factors associated with the psychological well-being among front-line nurses exposed to COVID-2019 in China: A predictive study. J Nurs Manag. 2020 Sep 5; doi: 10.1111/jonm.13146 32890453

[40] Mosheva M, Hertz‐Palmor N, Dorman Ilan S, Matalon N, Pessach IM, Afek A, et al. Anxiety, pandemic‐related stress and resilience among physicians during the covid‐19 pandemic. Depress Anxiety. 2020 Aug 12; 10.1002/da.23085 PMC7436709 32789945

[41] Chow SK, Francis B, Ng YH, Naim N, Beh HC, Ariffin MAA, et al. Religious Coping, Depression and Anxiety among Healthcare Workers during the COVID-19 Pandemic: A Malaysian Perspective. Healthc (Basel, Switzerland). 2021 Jan 15;9(1). doi: 10.3390/healthcare9010079 33467744 PMC7831030

[42] Lorente L, Vera M, Peiró T. Nurses´ stressors and psychological distress during the COVID-19 pandemic: The mediating role of coping and resilience. J Adv Nurs. 2020 Nov 19; 10.1111/jan.14695 PMC7753515 33210768

[43] Secer I, Ulas S, Karaman-Ozlu Z, Seçer İ, Ulaş S, Karaman-Özlü Z. The Effect of the Fear of COVID-19 on Healthcare Professionals’ Psychological Adjustment Skills: Mediating Role of Experiential Avoidance and Psychological Resilience. Front Psychol. 2020 Oct 21;11: 561536. doi: 10.3389/fpsyg.2020.561536 33192830 PMC7609966

[44] Jokić-Begić N, Lauri Korajlija A, Begić D. Mental Health of Psychiatrists and Physicians of Other Specialties in Early COVID-19 Pandemic: Risk ind Protective Factors. Psychiatr Danub. 2020 Sep;32(3–4): 536–548. doi: 10.24869/psyd.2020.536 33370764

[45] Dong Z-Q, Ma J, Hao Y-N, Shen X-L, Liu F, Gao Y, et al. The social psychological impact of the COVID-19 pandemic on medical staff in China: A cross-sectional study. Eur Psychiatry. 2020 Jun 1;63(1): e65. doi: 10.1192/j.eurpsy.2020.59 32476633 PMC7343668

[46] Britt TW, Shuffler ML, Pegram RL, Xoxakos P, Rosopa P, Hirsh E, et al. Job Demands and Resources among Healthcare Professionals during Virus Pandemics: A Review and Examination of Fluctuations in Mental Health Strain during COVID-19. Appl Psychol. 2020 Dec 2; doi: 10.1111/apps.12304 33362329 PMC7753503

[47] Shahrour G, Dardas LA. Acute stress disorder, coping self-efficacy and subsequent psychological distress among nurses amid COVID-19. J Nurs Manag. 2020 Oct;28(7): 1686–1695. doi: 10.1111/jonm.13124 32767827 PMC7436502

[48] Krok D, Zarzycka B. Risk Perception of COVID-19, Meaning-Based Resources and Psychological Well-Being amongst Healthcare Personnel: The Mediating Role of Coping. J Clin Med. 2020 Oct;9(10). 10.3390/jcm9103225 PMC7599885 33050068

[49] Balay-Odao EM, Alquwez N, Inocian EP, Alotaibi RS. Hospital Preparedness, Resilience, and Psychological Burden Among Clinical Nurses in Addressing the COVID-19 Crisis in Riyadh, Saudi Arabia. Front public Heal. 2021 Jan 8;8: 573932. 10.3389/fpubh.2020.573932 PMC7821043 33490012

[50] Yıldırım M, Arslan G, Özaslan A. Perceived Risk and Mental Health Problems among Healthcare Professionals during COVID-19 Pandemic: Exploring the Mediating Effects of Resilience and Coronavirus Fear. Int J Ment Health Addict. 2020 Nov 16; doi: 10.1007/s11469-020-00424-8 33223977 PMC7668285

[51] Pang Y, Fang H, Li L, Chen M, Chen Y, Chen M. Predictive factors of anxiety and depression among nurses fighting coronavirus disease 2019 in China. Int J Ment Health Nurs. 2021 Jan 24; doi: 10.1111/inm.12817 33491299 PMC8014285

[52] Chen J, Liu X, Wang D, Jin Y, He M, Ma Y, et al. Risk factors for depression and anxiety in healthcare workers deployed during the COVID-19 outbreak in China. Soc Psychiatry Psychiatr Epidemiol. 2021 Jan 10;56(1): 47–55. doi: 10.1007/s00127-020-01954-1 32914298 PMC7483060

[53] Sharma R, Seth S, Solanki HK, Mishra N, Srivastava A, Jakhar K. COVID-19 and Obstetrical Care: Coping With New Stress. CUREUS. 2020;12(12). doi: 10.7759/cureus.12116 33489531 PMC7808962

[54] Xiao H, Zhang Y, Kong D, Li S, Yang N. The Effects of Social Support on Sleep Quality of Medical Staff Treating Patients with Coronavirus Disease 2019 (COVID-19) in January and February 2020 in China. Med Sci Monit. 2020 Mar 5;26: e923549. doi: 10.12659/MSM.923549 32132521 PMC7075079

[55] Ni MY, Yang L, Leung CMC, Li N, Yao XI, Wang Y, et al. Mental Health, Risk Factors, and Social Media Use During the COVID-19 Epidemic and Cordon Sanitaire Among the Community and Health Professionals in Wuhan, China: Cross-Sectional Survey. JMIR Ment Heal. 2020 May 12;7(5): e19009. doi: 10.2196/19009 32365044 PMC7219721

[56] Woon LS-C, Sidi H, Nik Jaafar NR, Leong Bin Abdullah MFI. Mental Health Status of University Healthcare Workers during the COVID-19 Pandemic: A Post-Movement Lockdown Assessment. Int J Environ Res Public Health. 2020 Dec 8;17(24). doi: 10.3390/ijerph17249155 33302410 PMC7762588

[57] Tran V T, Nguyen HCHQHC, Pham V L, Nguyen MH, Nguyen HCHQHC, Ha TH, et al. Impacts and interactions of COVID-19 response involvement, health-related behaviours, health literacy on anxiety, depression and health-related quality of life among healthcare workers: a cross-sectional study. BMJ Open. 2020 Dec 7;10(12): e041394. doi: 10.1136/bmjopen-2020-041394 33293320 PMC7722826

[58] Young KP, Kolcz DL, O’Sullivan DM, Ferrand J, Fried J, Robinson K. Health Care Workers’ Mental Health and Quality of Life During COVID-19: Results From a Mid-Pandemic, National Survey. Psychiatr Serv. 2021 Feb 1;72(2): 122–128. doi: 10.1176/appi.ps.202000424 33267652

[59] Yörük S, Güler D. The relationship between psychological resilience, burnout, stress, and sociodemographic factors with depression in nurses and midwives during the COVID-19 pandemic: A cross-sectional study in Turkey. Perspect Psychiatr Care. 2021 Jan 26;57(1): 390–398. doi: 10.1111/ppc.12659 33103773

[60] Vagni M, Giostra V, Maiorano T, Santaniello G, Pajardi D. Personal Accomplishment and Hardiness in Reducing Emergency Stress and Burnout among COVID-19 Emergency Workers. Sustainability. 2020 Nov;12(21).

[61] Roslan NS, Yusoff MSB, Razak AA, Morgan K. Burnout Prevalence and Its Associated Factors among Malaysian Healthcare Workers during COVID-19 Pandemic: An Embedded Mixed-Method Study. Healthc (Basel, Switzerland). 2021 Jan 17;9(1). 10.3390/healthcare9010090 PMC7829836 33477380

[62] Manzano García G, Ayala Calvo JC, Manzano Garcia G, Ayala Calvo JC. The threat of COVID-19 and its influence on nursing staff burnout. J Adv Nurs. 2020 Nov 6; doi: 10.1111/jan.14642 33155716

[63] Soto-Rubio A, Giménez-Espert MDC, Prado-Gascó V. Effect of Emotional Intelligence and Psychosocial Risks on Burnout, Job Satisfaction, and Nurses’ Health during the COVID-19 Pandemic. Int J Environ Res Public Health. 2020 Oct 30;17(21). 10.3390/ijerph17217998 PMC7663663 33143172

[64] Ozdemir S, Kerse G. The Effects of COVID-19 Process on Health Care Workers: Analysing of the Relationships between Optimism, Job Stress and Emotional Exhaustion. Int Multidiscip J Soc Sci. 2020 Jul;9(2, SI): 178–201.

[65] Ramaci T, Barattucci M, Ledda C, Rapisarda V. Social Stigma during COVID-19 and its Impact on HCWs Outcomes. Sustainability. 2020 May 8;12(9): 3834.

[66] Hines SE, Chin KH, Glick DR, Wickwire EM. Trends in Moral Injury, Distress, and Resilience Factors among Healthcare Workers at the Beginning of the COVID-19 Pandemic. Int J Environ Res Public Health. 2021 Jan 9;18(2). 10.3390/ijerph18020488 PMC7826570 33435300

[67] Vagni M, Maiorano T, Giostra V, Pajardi D. Hardiness and Coping Strategies as Mediators of Stress and Secondary Trauma in Emergency Workers during the COVID-19 Pandemic. Sustainability. 2020 Sep;12(18).

[68] Chew QH, Chia FL-A, Ng WK, Lee WCI, Tan PLL, Wong CS, et al. Perceived Stress, Stigma, Traumatic Stress Levels and Coping Responses amongst Residents in Training across Multiple Specialties during COVID-19 Pandemic-A Longitudinal Study. Int J Environ Res Public Health. 2020 Sep 9;17(18). doi: 10.3390/ijerph17186572 32916996 PMC7559162

[69] Bruffaerts R, Voorspoels W, Jansen L, Kessler RC, Mortier P, Vilagut G, et al. Suicidality among healthcare professionals during the first COVID19 wave. J Affect Disord. 2021 Jan 11;283: 66–70. doi: 10.1016/j.jad.2021.01.013 33524660 PMC7832920

[70] Liao C, Guo L, Zhang C, Zhang M, Jiang W, Zhong Y, et al. Emergency stress management among nurses: A lesson from the covid‐19 outbreak in china–a cross‐sectional study. J Clin Nurs. 2020 Nov 19; doi: 10.1111/jocn.15553 33141483

[71] Vinkers CH, van Amelsvoort T, Bisson JI, Branchi I, Cryan JF, Domschke K, et al. Stress resilience during the coronavirus pandemic. Eur Neuropsychopharmacol. 2020 Jun;35(January): 12–16. doi: 10.1016/j.euroneuro.2020.05.003 32446705 PMC7211573

[72] Connor KM, Davidson JRT. Development of a new resilience scale: the Connor-Davidson Resilience Scale (CD-RISC). Depress Anxiety. 2003;18(2): 76–82. doi: 10.1002/da.10113 12964174

[73] Smith BW, Dalen J, Wiggins K, Tooley E, Christopher P, Bernard J. The brief resilience scale: assessing the ability to bounce back. Int J Behav Med. 2008;15(3): 194–200. doi: 10.1080/10705500802222972 18696313

[74] Hiebel N, Rabe M, Maus K, Peusquens F, Radbruch L, Geiser F. Resilience in Adult Health Science Revisited—A Narrative Review Synthesis of Process-Oriented Approaches. Front Psychol. 2021 Jun 3;12. doi: 10.3389/fpsyg.2021.659395 34149549 PMC8210849

[75] Pargament K, Feuille M, Burdzy D. The Brief RCOPE: Current Psychometric Status of a Short Measure of Religious Coping. Religions. 2011 Feb 22;2(1): 51–76.

[76] Moccia L, Janiri D, Pepe M, Dattoli L, Molinaro M, De Martin V, et al. Affective temperament, attachment style, and the psychological impact of the COVID-19 outbreak: an early report on the Italian general population. Brain Behav Immun. 2020 Jul;87(January): 75–79. 32325098 10.1016/j.bbi.2020.04.048 PMC7169930

[77] Wagerman SA, Bedikian A, Ross BS. Psychodynamic and sociopolitical predictors of COVID Distress and Gravity. Pers Individ Dif. 2021 Mar;171(January): 110506. doi: 10.1016/j.paid.2020.110506 33250549 PMC7680529

[78] Kunzler AM, Röthke N, Günthner L, Stoffers-Winterling J, Tüscher O, Coenen M, et al. Mental burden and its risk and protective factors during the early phase of the SARS-CoV-2 pandemic: systematic review and meta-analyses. Global Health. 2021 Dec 29;17(1): 34. doi: 10.1186/s12992-021-00670-y 33781283 PMC8006628

[79] Gilan D, Röthke N, Blessin M, Kunzler A, Stoffers-Winterling J, Müssig M, et al. Psychomorbidity, Resilience, and Exacerbating and Protective Factors During the SARS-CoV-2 Pandemic. Dtsch Aerzteblatt Online. 2020 Sep 18;117(28): 625–632. 10.3238/arztebl.2020.0625 PMC7817784 33200744

[80] Domes G, Schulze L, Böttger M, Grossmann A, Hauenstein K, Wirtz PH, et al. The neural correlates of sex differences in emotional reactivity and emotion regulation. Hum Brain Mapp. 2009 Dec 2;31(5): 758–769. 10.1002/hbm.20903 PMC6871188 19957268

[81] Kim SC, Rankin L, Ferguson J. Nurses’ mental health from early COVID‐19 pandemic to vaccination. J Nurs Scholarsh. 2021 Dec 30; doi: 10.1111/jnu.12760 34967492

