# Supplementary material for: The impact of the COVID-19 pandemic on the mental health of medical staff considering the interplay of pandemic burden and psychosocial resources—A rapid systematic review
Source: PLoS One. 2022 Feb 22;17(2):e0264290. doi: 10.1371/journal.pone.0264290 (PMC8863237; doi:10.1371/journal.pone.0264290)
Supplement: S1 Appendix — (PDF) [file pone.0264290.s002.pdf]

Appendix 1 – Summary of search terms

Population: ("healthcare worker*" OR "healthcare personnel*" OR "healthcare professional*" OR "medical professional*" OR "medical worker*" OR "medical staff*" OR "medical personnel*" OR "hospital personnel*" OR "hospital staff*" OR "hospital worker*" OR "hospital employee*" OR "medical employee*" OR "healthcare employee*" OR "health care worker*" OR "health care personnel*" OR "health care employee*" OR "health care staff*" OR "healthcare staff*" OR "allied health staff*" OR "allied health professional*" OR "allied health personnel*" OR "allied health worker*" OR "doctor*" OR "nurse*" OR "physician*" OR "clinician*" OR "clinical assistant*" OR "ward clerk*" OR "hospital volunteer*" OR "hospital administrator*" OR "medical practitioner*" OR "healthcare practitioner*" OR "health care practitioner*" OR "allied health practitioner*" OR "health worker*" OR "health professional*" OR "health practitioner*" OR "health employee*" OR "health staff*" OR "emergency department staff*" OR "icu staff*" OR "intensive care unit staff*" OR "infectious disease unit*" OR "infectious disease ward*" OR "health care personnel" OR "health personnel")

Condition: ("2019-ncov" OR "coronavirus" OR "covid19" OR "covid" OR "covid-19" OR "ncov" OR "severe acute respiratory syndrome*" OR "sars-cov-2" OR "sars" OR "sars-related coronavirus" OR "coronavirus disease 2019")

Outcome (Pandemic burden/Mental health): ("stressor*" OR "stress factor*" OR "risk factor*" OR "load factor*" OR "burden*" OR "load" OR "burdensome factor*" OR "strain" OR "psychological strain" OR "psychosocial strain" OR "loading factor*" OR "work-life balance" OR "Effort-reward imbalance" OR "ERI" OR "psychosocial burden" OR "psychological burden" OR "inter-role conflict" OR "interrole conflict" OR "work-life balance" OR "workload" OR "concern*" OR "moral distress" OR "moral conflict" OR "mental health" OR "wellbeing" OR "psychological distress" OR "emotional stress" OR "Impact of event scale" OR "IES" OR "quality of life" OR "PHQ*" OR "Patient Health Questionnaire" OR "psychological symptom*" OR "psychological impact" OR "psychiatric symptom*" OR "depression" OR "depressive symptom*" OR "anxiety" OR "posttraumatic stress" OR "post-traumatic burden" OR "post-traumatic stress" OR "posttraumatic burden" OR "burnout" OR "Burn-out" OR "Maslach Burnout Inventory" OR "MBI")

Outcome (Resources): ("resilience" OR "resiliency" OR "psychosocial resources" OR "psychological resources" OR "spiritual*" OR "spiritual wellbeing" OR "social support" OR "optimism" OR "protective factor*" OR "coping" OR "active coping")

Search strategy adapted for PubMed

(("healthcare worker*"[All Fields] OR "healthcare personnel*"[All Fields] OR "healthcare professional*"[All Fields] OR "medical professional*"[All Fields] OR "medical worker*"[All Fields] OR "medical staff*"[All Fields] OR "medical personnel*"[All Fields] OR "hospital personnel*"[All Fields] OR "hospital staff*"[All Fields] OR "hospital worker*"[All Fields] OR "hospital employee*"[All Fields] OR "medical employee*"[All Fields] OR "healthcare employee*"[All Fields] OR "health care worker*"[All Fields] OR "health care personnel*"[All Fields] OR "health care employee*"[All Fields] OR "health care staff*"[All Fields] OR "healthcare staff*"[All Fields] OR "allied health staff*"[All Fields] OR "allied health professional*"[All Fields] OR "allied health personnel*"[All Fields] OR "allied health worker*"[All Fields] OR "doctor*"[All Fields] OR "nurse*"[All Fields] OR "physician*"[All Fields] OR "clinician*"[All Fields] OR "clinical assistant*"[All Fields] OR "ward clerk*"[All Fields] OR "hospital volunteer*"[All Fields] OR "hospital administrator*"[All Fields] OR "medical practitioner*"[All Fields] OR "healthcare practitioner*"[All Fields] OR "health care practitioner*"[All Fields] OR "allied health practitioner*"[All Fields] OR "health worker*"[All Fields] OR "health professional*"[All Fields] OR "health practitioner*"[All Fields] OR "health employee*"[All Fields] OR "health staff*"[All Fields] OR "emergency department staff*"[All Fields] OR "icu staff*"[All Fields] OR "intensive care unit staff*"[All Fields] OR "infectious disease unit*"[All Fields] OR "infectious disease ward*"[All Fields] OR "health care personnel"[All Fields] OR "health personnel"[All Fields]) AND ("2019-ncov"[All Fields] OR "coronavirus"[All Fields] OR "covid19"[All Fields] OR "covid"[All Fields] OR "covid-19"[All Fields] OR "ncov"[All Fields] OR "severe acute respiratory syndrome*"[All Fields] OR "sars-cov-2"[All Fields] OR "sars"[All Fields] OR "sars-related coronavirus"[All Fields] OR "coronavirus disease 2019"[All Fields]) AND ("stressor*"[All Fields] OR "stress factor*"[All Fields] OR "risk factor*"[All Fields] OR "load factor*"[All Fields] OR "burden*"[All Fields] OR "load"[All Fields] OR "burdensome factor*"[All Fields] OR "strain"[All Fields] OR "psychological strain"[All Fields] OR "psychosocial strain"[All Fields] OR "loading factor*"[All Fields] OR "work-life balance"[All Fields] OR "Effort-reward imbalance"[All Fields] OR "ERI"[All Fields] OR "psychosocial burden"[All Fields] OR "psychological burden"[All Fields] OR "inter-role conflict"[All Fields] OR "interrole conflict"[All Fields] OR "work-life balance"[All Fields] OR "workload"[All Fields] OR "concern*"[All Fields] OR "moral distress"[All Fields] OR "moral conflict"[All Fields] OR "mental health"[All Fields] OR "wellbeing"[All Fields] OR "psychological distress"[All Fields] OR "emotional stress"[All Fields] OR "Impact of event scale"[All Fields] OR "IES"[All Fields] OR "quality of life"[All Fields] OR "phq"[All Fields] OR "Patient Health Questionnaire"[All Fields] OR "psychological symptom*"[All Fields] OR "psychological impact"[All Fields] OR "psychiatric symptom*"[All Fields] OR "depression"[All Fields] OR "depressive symptom*"[All Fields] OR "anxiety"[All Fields] OR "posttraumatic stress"[All Fields] OR ("post traumatic"[All Fields] AND ("burden"[All Fields] OR "burdened"[All Fields] OR "burdening"[All Fields] OR "burdens"[All Fields])) OR "post-traumatic stress"[All Fields] OR ("posttraumatic"[All Fields] AND ("burden"[All Fields] OR "burdened"[All Fields] OR "burdening"[All Fields] OR "burdens"[All Fields])) OR "burnout"[All Fields] OR "Burn-out"[All Fields] OR "Maslach Burnout Inventory"[All Fields] OR "MBI"[All Fields]) AND ("resilience"[All Fields] OR "resiliency"[All Fields] OR "psychosocial resources"[All Fields] OR "psychological resources"[All Fields] OR "spiritual*"[All Fields] OR "spiritual wellbeing"[All Fields] OR "social support"[All Fields] OR "optimism"[All Fields] OR "protective factor*"[All Fields] OR "coping"[All Fields] OR "active coping"[All Fields])) AND ((english[Filter] OR german[Filter]) AND (2019:2021[pdat]))
