# Supplementary material for: The impact of the COVID-19 pandemic on the mental health of medical staff considering the interplay of pandemic burden and psychosocial resources—A rapid systematic review
Source: PLoS One. 2022 Feb 22;17(2):e0264290. doi: 10.1371/journal.pone.0264290 (PMC8863237; doi:10.1371/journal.pone.0264290)
Supplement: S2 Appendix — (PDF) [file pone.0264290.s003.pdf]

Abbreviation	Instrument
AWS	Areas of Worklife Scale
ASA	Adulthood Separation Anxiety
BES-A	Adaptation of the<br>Basic Empathy Scale - short version
BDI	Beck depression inventory
BRCOPE	Brief Coping Orientation to Problems Experienced
BRCS	Brief Resilience Coping Scale
BRS	Brief Resilience Scale
BSI-18	Brief Symptom Inventory-18
CAGE-AID	Substance Abuse Screening Tool
CBI	Copenhagen Burnout Inventory
CD-RISC	Connor-Davidson Resilience Scale
CESQT	Questionnaire for the Assessment of Workplace Burnout Syndrome
CMBI	15-item Chinese version of Maslach Burnout Inventory
COPM	Canadian Occupational Performance Measure
C-SSRS	Columbia-Suicide Severity Rating Scale
CSES-SF	Coping Self-Efficacy Scale
DASS-21	Depression Anxiety Stress Scales-21
DD	Depersonalization / Derealization inventory by Cox and Swinson (2002)
DERS-18	Difficulties in Emotion Regulation scale
DES	Dissociative Experiences Scale by Bernstein and Putnam
DRS-15	Dispositional Resilience Scale-15
ESQ	Emergency Stress Questionnaire
FEWS	Frankfurt Emotional Work Scale
GAD	General anxiety disorder scale
GAD-2	Generalized Anxiety Disorder 2
GAD-7	General Anxiety Disorder–7
GHQ-12	General Health Questionnaire-12
GHQ-28	General Health Questionnaire-2
GSES	General Self-Efficacy Scale
HADS	Hospital Anxiety and Depression Scale by Zigmond and Snaith
HEI	Huaxi Emotional-Distress Index
HRQoL	Health-related quality of life (RAND)
IES	Impact of Event Scale
IES-6	Impact of Event Scale-6
ISI	Insomnia Severity Index

JCQ	Job Content Questionnaire
JSS	Job Stress scale (by Sosik and Goldshalk (2000)
MBI	Maslach burnout inventory
MBI-HSS	Maslach Burnout Inventory Human Service Survey
MIES	Moral Injury Events Scale
MOS-SSS	Medical Outcomes Study Social Support Survey
MSPSS	Multidimensional Scale of Perceived Social Support
NSS	Nurses Stress Scale by Gray-Toft and Anderson
OLBI	Oldenburg Burnout Inventory
OPS	Optimism-Pessimism Scale (by Caliskan and Uzunkol (2018)
PCL-5	Post-traumatic stress disorder checklist
PC-PTSD	Primary Care Post-traumatic Stress Disorder Screen
PFI	Stanford Professional Fulfilment Index
PHQ-9	Patient Health Questionnaire–9
PROMIS	Patient-Reported Outcomes Measurement Information System
ProQOL	Professional Quality of Life Scale
PSQI	Pittsburgh Sleep Quality Index
PSQI	Pittsburgh Sleep Quality Index by Buysse et al.
PSS	Perceived stress scale
PSSS	perceived social support scale
PWBI	Mayo Clinic<br>Physician Well-Being Index
RS-14	14-Item Resilience Scale
RSA	Resilience scale for adults
RSES	Rosenberg Self-Esteem Scale
SAS	Self-Rating Anxiety Scale
SASRQ / SASR	Stanford Acute Stress Reaction Questionnaire
SCSQ	Simplified coping style questionnaire
SDQ	Strengths and Difficulties Questionnaire
SOS	Stress overload Scale
SSRS	Social Support Rate Scale
STAIsv	State-Trait Anxiety Inventory by Marteau and Bekker
STSS	Secondary Traumatic Stress Scale

SWEMWBS	Short Warwick Edinburgh Mental Wellbeing Scale
SWLS	Satisfaction with Life Scale
TCSQ	20-item Trait Coping Style Questionnaire
TMMS-24	Trait Meta-Mood Scale
UNIPSICO Battery	Unidad de Investigación Psicosocial de La Conducta Organizacional Battery
WRQOL	TheWork-Related Quality of Life Scale
	Copenhagen Burnout Inventory Scale-19 items
